# Whole exome sequencing and polygenic assessment of a Swedish cohort with severe developmental language disorder

**DOI:** 10.1007/s00439-023-02636-z

**Published:** 2024-02-01

**Authors:** Ashraf Yahia, Danyang Li, Sanna Lejerkrans, Shyam Rajagopalan, Nelli Kalnak, Kristiina Tammimies

**Affiliations:** 1 Center of Neurodevelopmental Disorders (KIND), Centre for Psychiatry Research, Department of Women’s and Children’s Health, Karolinska Institutet, Region Stockholm, Stockholm, Sweden; 2 Astrid Lindgren Children’s Hospital, Karolinska University Hospital, Region Stockholm, Stockholm, Sweden; 3 Social, Genetic and Developmental Psychiatry Centre, King’s College London, London, UK; 4 Institute of Bioinformatics and Applied Biotechnology, Bengaluru, India; 5 Department of Speech-Language Pathology, Helsingborg Hospital, Helsingborg, Sweden

## Abstract

Developmental language disorder (DLD) overlaps clinically, genetically, and pathologically with other neurodevelopmental disorders (NDD), corroborating the concept of the NDD continuum. There is a lack of studies to understand the whole genetic spectrum in individuals with DLD. Previously, we recruited 61 probands with severe DLD from 59 families and examined 59 of them and their families using microarray genotyping with a 6.8% diagnostic yield. Herein, we investigated 53 of those probands using whole exome sequencing (WES). Additionally, we used polygenic risk scores (PRS) to understand the within family enrichment of neurodevelopmental difficulties and examine the associations between the results of language-related tests in the probands and language-related PRS. We identified clinically significant variants in four probands, resulting in a 7.5% (4/53) molecular diagnostic yield. Those variants were in *PAK2*, *MED13*, *PLCB4*, and *TNRC6B*. We also prioritized additional variants for future studies for their role in DLD, including high-impact variants in *PARD3* and *DIP2C*. PRS did not explain the aggregation of neurodevelopmental difficulties in these families. We did not detect significant associations between the language-related tests and language-related PRS. Our results support using WES as the first-tier genetic test for DLD as it can identify monogenic DLD forms. Large-scale sequencing studies for DLD are needed to identify new genes and investigate the polygenic contribution to the condition.

**Supplementary Information:**

The online version contains supplementary material available at 10.1007/s00439-023-02636-z.

## Introduction

Developmental language disorder (DLD) and other neurodevelopmental disorders (NDD) share many symptoms and signs (Nitin et al. 2022; Plug et al. 2021). DLD is a neurodevelopmental communication disorder characterized by persistent deficits in receptive and expressive language not attributable to other biomedical conditions and results in functional, psychological, social, and/or occupational limitations (American Psychiatric Association 2022; Bishop et al. 2016). DLD can co-occur with other NDD, complicating their clinical and etiological disentanglement (Nitin et al. 2022). DLD remains underdiagnosed and understudied despite its ~7% prevalence (Calder et al. 2022; McGregor 2020; Tomblin et al. 1997). Growing evidence suggests that genetic factors have a large impact on the etiology of DLD (Mountford et al. 2022). Some environmental risk factors, such as maternal smoking during pregnancy, have also been suggested (Calder et al. 2022).

Monogenic/genomic DLD forms, although rare, are apt to provide insight into the biological pathways associated with DLD. In this study, we used the term genomic DLD when referring to DLD caused by copy-number variants (CNVs). An increasing number of high-impact copy CNVs and rare variants have been identified in a proportion of DLD individuals (Chen et al. 2017a, b; Eising et al. 2019; Kalnak et al. 2018; Mountford et al. 2022; Plug et al. 2021; Villanueva et al. 2015). The recent advances in sequencing technologies and the availability of population-level variations data have enabled genetic diagnosis in DLD and the discovery of new genes implicated in the condition (Mountford et al. 2022).

Disentangling the polygenic contribution in DLD is lagging compared to the monogenic forms. A recent genome-wide association study (GWAS) identified a robust single-nucleotide polymorphism (SNP) heritability of five reading- and/or language-related traits, not including DLD, that explained 13–26% of trait variability (Eising et al. 2022).

Previously, we reported an enrichment of neurodevelopmental difficulties in families of 59 probands with severe DLD (Kalnak et al. 2012, 2014). In a subsequent study, we investigated those 59 probands and their families using array genotyping (Kalnak et al. 2018). We reported clinically significant CNVs in four probands, resulting in a 6.8% molecular diagnostic yield. Although we identified rare CNVs in some siblings and parents, a significant association with CNV burden did not explain the aggregation of NDD within the families (Kalnak et al. 2018). In the current study, we investigated 53 out of the 59 probands using whole exome sequencing (WES) to identify rare monogenic conditions. We report the high-impact variants identified in genes previously known to be associated with monogenic DLD/DLD-related disorders, highlighting the diagnostic utility of WES in DLD. Furthermore, we prioritized candidate genes for future studies. We also explored the possible association between the performance of individuals with DLD on selected language-related tests and their corresponding polygenic scores, and the potential of PRS to explain the enrichment of neurodevelopmental difficulties in the families.

## Methods

### Recruitment of participants

Sixty-one DLD probands aged 8–12 years were recruited from 14 school language units for children with severe DLD as described before (Kalnak et al. 2012, 2014, 2018). Admission to these schools requires pre-assessments by a speech-language pathologist, a psychologist, and a teacher, and is usually performed at the age of 5 or 6 years. To be admitted, each child had to have DLD as the primary diagnosis, i.e., excluding autism spectrum disorder (ASD) and intellectual disability (ID). Children with dyslexia or attention-deficit hyperactivity disorder (ADHD) could be admitted if they co-occurred with DLD. Children whose DLD status was confirmed by the school to still be the primary diagnosis at the time of participation were included.

### Inclusion and exclusion criteria

As described previously (Kalnak et al. 2012, 2014, 2018; Kalnak and Sahlén 2022), our inclusion criteria were: (1) DLD at the time of school admission and not questioned by the school up to the time of recruitment to our study at the age of 8–12 years. (2) Normal nonverbal IQ at admission to school (3) Normal vision and hearing. (4) Monolingual Swedish speaking. We included only monolingual Swedish-speaking children to minimize the risk of bilingual effects on the language tests which were all administered in Swedish. Adopted children were excluded from the study. We also excluded probands without available high-quality DNA from this current study.

### Interviews and assessments

The probands were assessed with a broad linguistic and cognitive test battery by a speech-language pathologist, as described previously (Kalnak et al. 2018). Herein, we used the results from the nonword repetition task (NWR), the Raven’s colored progressive matrices (CPM9), and the test of word-reading efficiency (TOWRE). NWR standard deviation units (*z*-scores) were calculated based on whole-word accuracy (NWR_binary; the binary outcome of either a positive or a negative response), numbers of syllabi (NWR_length), and percentage of correct consonants (NWR_PCC). TOWRE z-scores were based on the number of accurate decoding over time of words (TOWRE_words) and nonwords (TOWRE_nonwords). We also compared the performance of the probands with a monogenic diagnosis identified in the current or the previous study (Kalnak et al. 2018) on CPM9, NWR_binary, NWR_length, NWR_PCC, TOWRE_words, and TOWRE_nonwords.

To investigate the polygenic contribution of other neurodevelopmental diagnoses and traits/difficulties within these families, we also categorized each participant, including parents and siblings, whether they were reported with any kind of neurodevelopmental (1) diagnoses, such as language disorder, reading impairments, autism spectrum disorder, stuttering, ID, ADHD, cleft palate, congenital impairment of hearing or vision, or (2) difficulties with language, learning, social communication, and attention. These were based on family history interviews with the parents (Kalnak et al. 2012).

### Sample collection and DNA extraction

Saliva samples were collected from the participants using the Oragene DNA OG-500 collection tubes (DNA Genotek, Inc., Ottawa, Ontario, Canada). DNA was extracted according to the prepIT.L2P manual protocol provided by the manufacturer. DNA quality was checked using agarose gel electrophoresis and NanoDrop spectrophotometer.

### Whole exome sequencing and variant prioritization

Fifty-three out of the 59 participants with available DNA were examined using WES. Four samples were excluded due to degraded DNA and two failed during library preparation. WES was performed on NovaSeq 6000 using paired end reads at the Clinical Genomics Stockholm core facility, Karolinska Institutet, and Science for Life Laboratory, Stockholm, Sweden. Library preparation and WES data processing up to the generation of variant call format files (VCF) were described previously (Li et al. 2022). In short, Twist Human Core Exome v1.3 Enrichment Kit (Twist Bioscience) was used for library preparation with some modifications: xGen Dual Index UMI adapters [6-nucleotide unique molecular identifiers (UMI), 0.6 mM, Integrated DNA Technologies] were used for the ligation, and xGen Library Amp Primer (2 mM, Integrated DNA Technologies) was used for PCR amplification (10 cycles). Target enrichment was performed in a multiplex fashion with a library amount of 187.5 ng (8-plex). The libraries were hybridized to Exome probes v1.3 (Twist Bioscience), xGen Universal Blockers—TS Mix (Integrated DNA Technologies), and COT Human DNA (Life Technologies) for 16–19 h. The post-capture PCR was performed with xGen Library Amp Primer (0.5 mM, Integrated DNA Technologies) for eight cycles. Quality control was performed with the Qubit dsDNA HS assay (Invitrogen) and TapeStation HS D1000 assay (Agilent). Demultiplexing was done using Casava v2.20. The depth and coverage were assessed relative to the capture kit and the exome-coding regions of hg19 using Samtools v1.17. The average coverage of the hg19 whole exome was 79% (the range was 76–81%), 75% (70–77%), and 73% (65–74%) at 10×, 20×, and 30×, respectively. The average coverage of the capture kit was 99% (87–99.7%; only two samples were below 99%, we did not identify a candidate variant in those two samples) at 30× and the mean depth was 188.

The reads were aligned against the GRCh37 reference genome using Burrows Wheel Aligner (Li and Durbin 2009). We used VarAFT to annotate the VCF files and prioritize/filter the variants (Desvignes et al. 2018). First, we selected exonic and probable splice-site variants that were rare in gnomAD v2.1 general and European populations (minor allele frequency < 0.001 for recessive model and <0.000001 for dominant/de novo model), predicted as damaging/possibly damaging/probably damaging by in silico tools (Sift (Ng and Henikoff 2003), Polyphen2 HumDiv, and Polyphen2 HumVar (Adzhubei et al. 2010)), and located in brain-expressed genes. In the subsequent filters, we selected only high-impact variants in genes that fitted the postulated inheritance models in each family and were considered essential for brain function.

We regarded a variant as a high-impact variant if it was nonsense, start-loss, ±2 splice-site, or a frameshift variant; or if it was classified by ClinVar as pathogenic/likely pathogenic variant. Genes were considered essential for brain function if when mutated cause a central nervous system disease (CNS; searched in OMIM and PubMed databases) and/or if they were constrained genes (damaging variants of them are removed by natural selection). Additionally, genes not reported before to cause a CNS disease had to have evidence from the scientific literature supporting their role in the CNS. We considered dominant genes constrained if their gnomAD v2.1 probability of being loss-of-function intolerant (pLI) was ≥0.9. We considered recessive genes constrained if the number of homozygotes with loss-of-function variants in gnomAD v2.1 general population was zero. Furthermore, we searched the scientific literature using PubMed database by entering the name/s of the gene in the search field.

Supplementary Fig. S1 shows the filters we used for the dominant/de novo model and the average number of variants that remained after each filter. We used similar filters for recessive models (homozygous and compound heterozygous), with 0.001 as a frequency cutoff.

Candidate variants were sent for Sanger sequencing for segregation analysis. Primers were designed using Primer3Plus (Untergasser et al. 2007). Sanger sequencing was performed at Eurofins Genomics Europe Sequencing GmbH, Köln, Germany, and viewed using SnapGene Viewer. We submitted all the candidate novel genes to the GeneMatcher platform (Sobreira et al. 2015). Variants in genes known to cause NDD were classified according to the American College of Medical Genetics (ACMG) criteria (Richards et al. 2015) and amending recommendations for PVS1 and PP3/BP4 criteria (Abou Tayoun et al. 2018; Pejaver et al. 2022). For each prioritized variant, we further checked if it meets the amended recommendation for PVS1 using AutoPVS1 (Xiang et al. 2020). We confirmed trio status of the parents and the proband from genome-wide SNP array data, when available, using “--kinship” command of KING v2.2.9 to aid ACMG variants classification.

GnomAD, PubMed, ClinVar, and GeneMatcher platforms/databases were accessed November 22nd, 2023.

### Genotyping and imputation

Genotyping using Genome-Wide SNP Array 6.0 (Affymetrix, Santa Clara, California) of the 59 families has been described earlier (Kalnak et al. 2018). Calling of the genotypes was performed using Affymetrix Analysis Power Tools (APT) software version APT-2.11.6. The average per sample call rate was >97%. APT output genotyping file was converted to VCF using affy2vcf plugin of BCFtools (Li 2011). Quality control was performed using PLINK 1.9 (Chang et al. 2015). Variants or individuals with missingness > 2% (tightened to > 1% before PRS calculation) were filtered. Variants not in Hardy–Weinberg equilibrium (*p* < 1 × 10^−6^) were also filtered. Individuals with heterozygosity rate >±3 standard deviations from the samples’ heterozygosity rate mean were removed. Discrepancies between reported and biological sex were checked using the “–check-sex” PLINK command (X chromosome homozygosity estimate for males > 0.8 and for females < 0.2). Checking population stratification was performed using the multidimensional scaling (MDS) approach anchored to phase 3 of the 1000 genome project as described by Marees et al. (Marees et al. 2018). We checked for any strand issues, any problematic SNPs, and references correspondence before anchoring. MDS component scores were generated using “--cluster --mds-plot” PLINK command. We visualized the first two principal components to check for outliers (Supplementary Fig. S2). Minor allele frequency was set at 5%. Out of 180 individuals and 907,343 variants, 165 individuals and 490,971 variants passed our quality control filters with a total genotyping rate of 99.7%. Six individuals were filtered due to high missingness and nine due to excess heterozygosity. Phasing and imputation were performed based on phase 3 of the 1000 genome project using the default parameters of SHAPEIT4 (Delaneau et al. 2019) and IMPUTE5 (Rubinacci et al. 2020), respectively.

We previously described calling CNVs from this same Genome-Wide SNP array data of the 59 families using three algorithms: PennCNV-Affy (August 2016 version), Chromosome Analysis Suite (ChAS) software v.3.1 (Affymetrix), and Partek Genomics Suite software version 6.6 (Partek Inc., St. Louis, Missouri) (Kalnak et al. 2018). In short, the CNV calls were mapped using GRCh37/hg19 coordinates and annotated using ANNOVAR. Only CNVs called by at least two of the algorithms with ≥50% reciprocal overlap were considered, excluding those that spanned ≥50% with centromeres or telomeres, <10 consecutive probes and were <10 kb in size (Kalnak et al. 2018).

### Polygenic risk scores’ calculations and analysis

We selected five GWASs on European-ancestry individuals as base data sources to calculate PRS for specific developmental traits to test possible polygenic overlap with DLD. These traits included ASD (46,350 individuals) (Grove et al. 2019), ADHD (51,568 individuals) (Demontis et al. 2023), educational attainment (765,283 individuals) (Okbay et al. 2022), cognitive performance (257,828 individuals) (Lee et al. 2018), reading- and language-related skills, and performance IQ (Eising et al. 2022). The included reading- and language-related skills were word reading (27,180 individuals), nonword reading (16,746 individuals), nonword repetition (12,828 individuals), phoneme awareness (12,411 individuals), and spelling (17,278 individuals). PRS was calculated using the default parameters of PRS-CS (Ge et al. 2019) (PARAM_A = 1, PARAM_B = 0.5, PARAM_PHI = 0.01, MCMC_ITERATIONS = 1000, MCMC_BURNIN = 500, and MCMC_THINNING_FACTOR = 5). Polygenic transmission disequilibrium was tested in 30 individuals from 20 families using ptdt tool (Weiner et al. 2017). The first ten principal components of ancestry (PC1–10) were included as covariates in the association analysis between the selected language-related clinical measures and PRS. We calculated the first ten principal components using “--pca” PLINK command.

### Statistical analysis

We compared the performance of the probands with a monogenic/genomic diagnosis identified in the current and the previous study (Kalnak et al. 2018) to the probands without any genetic diagnosis on CPM9, NWR_binary, NWR_length, NWR_PCC, TOWRE_words, and TOWRE_nonwords tasks using an unpaired student *t* test.

We fitted a generalized estimating equation (GEE) logistic regression model in R version 4.1.1 using the geeglm() function from the geepack package (Halekoh et al. 2006) to test associations between the PRS for ASD, ADHD, educational attainment, and cognitive performance and the neurodevelopmental phenotypes in the families. We added sex and the first ten principal components (PC1–10) as predictor variables, assuming an independent correlation structure and controlling for the family status.

The associations between six quantitative language and reading skill measures tested in the DLD probands and the corresponding PRS values were done using a linear regression with lm() function in R. The models were adjusted for age, sex, diagnosis with a monogenic condition/genomic syndrome, and PC1–10. CPM9 was tested against cognitive performance PRS and performance IQ PRS, each in a separate model. NWR scores were tested against nonword repetition PRS, and TOWRE scores were tested against word reading and nonword reading PRS, in separate models.

## Results

### Diagnostic yield of WES and inheritance patterns

**Fig. 1:**
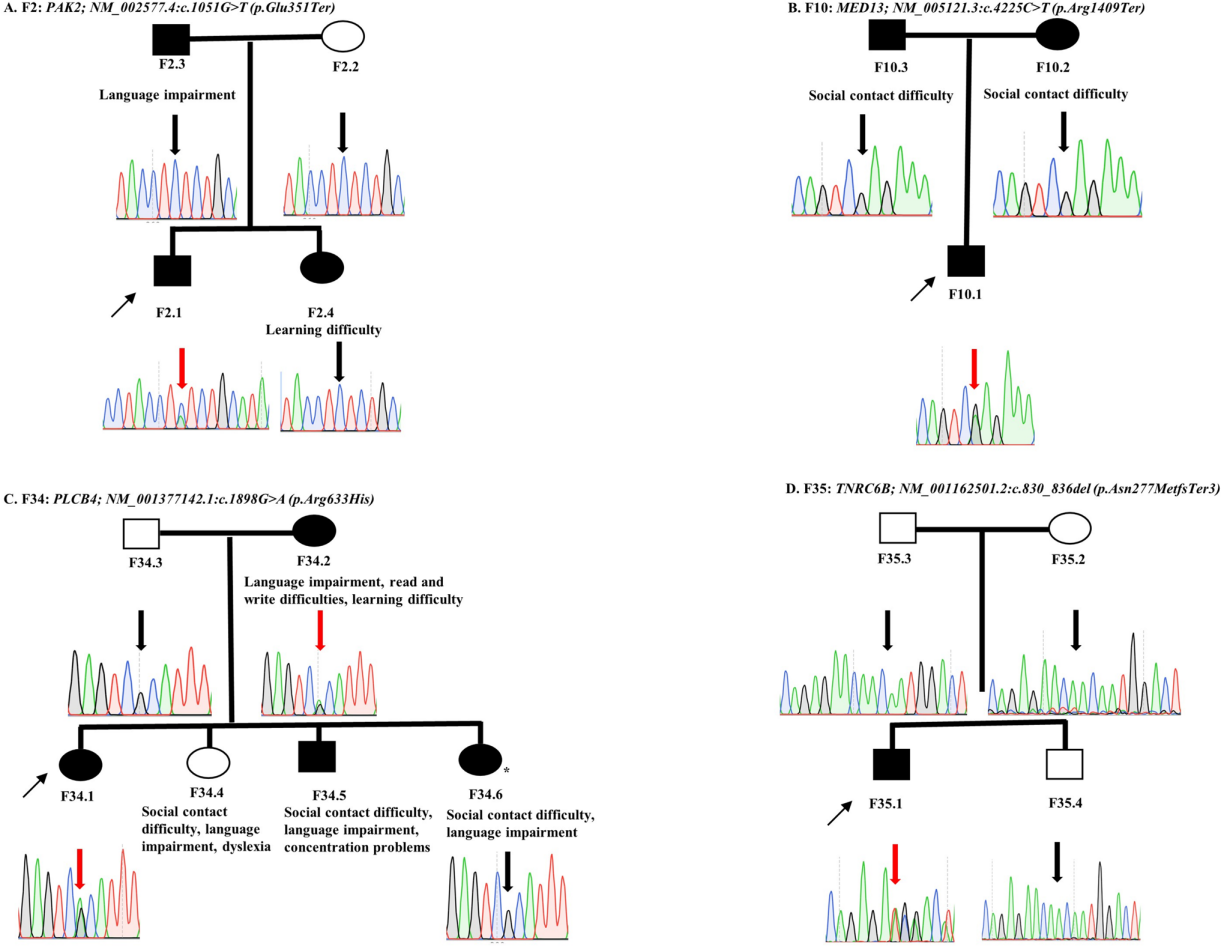
Segregation analysis of the variants identified in genes known to cause developmental language disorders. **A**–**D** Thin arrows point to the proband in each family, thick red arrows point to culprit variants, and black arrows point to wild-type variants, circles depict females, squares depict males, filled circles and squares depict patients, and unfilled circles and squares depict controls. DLD, developmental language disorders. We previously identified a 3.5 Mb deletion of Xp22.31-p22.33 in F34.6, highlighted with “*” sign

Among the 53 probands, we identified four pathogenic/likely pathogenic variants in genes previously known to cause DLD or other related NDD in four probands/families, achieving a molecular diagnostic success rate of 7.5% (4/53) (Table 1). Three of the variants were de novo variants and one was inherited from an apparently affected father (Fig. 1). The four variants were pathogenic/likely pathogenic according to the ACMG criteria (Richards et al. 2015) and the amendments for PVS1 and/or PP3/BP4 criteria (Abou Tayoun et al. 2018; Pejaver et al. 2022). A variant of uncertain significance (NM_001005271.3:c.2327A>G (p.Asp776Gly)) in *CHD3* was detected in family F60 (Supplementary materials) and was not included as a positive diagnosis when we calculated the molecular diagnostic success rate. KING kinship inference was as expected in the families.

**Table 1:** The variants identified in genes previously known to cause developmental language disorders or related phenotypes

Individual	Gene	Variant type	Variant	Genotype/inheritance	gnomAD frequency	ACMG classification	ACMG criteria	Previously known phenotype
*High impact variants in genes known to cause NDD in humans*								
F2.1	*PAK2*	Nonsense	NM_002577.4:c.1051G>T (p.Glu351Ter)	Heterozygous/de novo	Absent	Pathogenic	PVS1^a^, PS2^b^, and PM2	Knobloch syndrome 2 (OMIM 618458) and ASD-like syndrome
F10.1	*MED13*	Nonsense	NM_005121.3:c.4225C>T (p.Arg1409Ter)	Heterozygous/de novo	Absent	Likely pathogenic	PVS1^a^, and PM2	Autosomal dominant intellectual developmental disorder 61 (OMIM 618009)
F34.1	*PLCB4*	Missense	NM_000933.4:c.1862G>A (p.Arg621His)	Heterozygous/dominant	Absent	Pathogenic	PS1, PS3, PM1, PM2, PM5, PP2, and PP3_supporting^c^	Auriculocondylar syndrome 2 (OMIM 614669)
F35.1	*TNRC6B*	Frameshift deletion	NM_001162501.2:c.830_836del (p.Asn277MetfsTer3)	Heterozygous/de novo	Absent	Pathogenic	PVS1^a^, PS2^b^, and PM2	Global developmental delay with speech and behavioral abnormalities (OMIM 619243)

ACMG, the American College of Medical Genetics and the Association for Molecular Pathology; PVS1, null variant in a gene where loss of function is a known disease mechanism; PS2, de novo (both maternity and paternity confirmed) in a patient with the disease and no family history; PM2, absent or have low frequency in gnomAD public database; PS1, same amino acid change as a previously established pathogenic variant regardless of nucleotide change; PS3, well-established in vitro or in vivo functional studies supportive of a damaging effect on the gene or gene product, PM1, located in a mutational hot spot and/or critical and well-established functional domain without benign variation; PM5, novel missense change at an amino acid residue where a different missense change determined to be pathogenic has been seen before; PP2, missense variant in a gene that has a low rate of benign missense variation and in which missense variants are a common mechanism of disease; PP3, multiple lines of computational evidence support a deleterious effect; OMIM, online inheritance in man

^a^PVS1 variants met Abou Tayoun et al. (2018) recommendation and were validated in silico using AutoPVS1 tool

^b^Kinship tested using KING software

^c^PP3_supporting based on Pejaver et al. 2022 recommendation for ACMG criteria update (REVEL score was 0.726)

### Pathogenic/likely pathogenic variants in genes known to cause DLD/NDD in human

In family F2, we identified the de novo variant NM_002577.4(PAK2):c.1051G>T (p.Glu351Ter) in the proband F2.1 (Fig. 1A). Heterozygous mutations in *PAK2* were only reported before in a Chinese ASD individual (Wang et al. 2018) and in two New Zealand siblings with a variable combination of retinal detachment, vitreous abnormalities, severe developmental delay, incomprehensible speech, ASD, interstitial parenchymal pulmonary changes on chest X-rays, and enamel hypoplasia (OMIM 618458) (Antonarakis et al. 2021).

In family F10, we identified the de novo nonsense variant affecting *MED13* [NM_005121.3(MED13):c.4225C>T (p.Arg1409Ter)] (Fig. 1B). Heterozygous mutations in *MED13* cause intellectual developmental disorder type 61 (OMIM 618009) (Snijders Blok et al. 2018). The clinical presentations of individuals with pathogenic *MED13* variants include variable combinations of NDD, dysmorphic features, and epilepsy among others (Snijders Blok et al. 2018; Trivisano et al. 2022).

In family F34, we identified the missense variant NM_001377142.1(PLCB4):c.1898G>A (p.Arg633His) (rs397514481) in the proband and her mother (Fig. 1C). The variant was absent from the gnomAD v4.0 database, had a CADD score of 35, was classified as pathogenic in ClinVar database by six submitters (VCV000031639.6, accessed in 28/02/2023), and was validated by functional studies (Kanai et al. 2022). Recessive and dominant mutations in *PLCB4* cause auriculocondylar syndrome 2 (OMIM 614669) (Gordon et al. 2013; Rieder et al. 2012). Dominant mutations, including rs397514481, cause the disease through dominant-negative effects on the endothelin receptor type A-Gq/11 pathway important for forming lower jaw and middle ear structures during embryonic development (Kanai et al. 2022). Auriculocondylar syndrome 2 is characterized by craniofacial anomalies and other less frequent features, including speech delay (Li et al. 2023). Variable expressivity and incomplete penetrance have also been observed in auriculocondylar syndrome 2 (Vegas et al. 2022). While the proband only had severe DLD, his mother had reading and writing difficulties, language impairment, and learning difficulties. The proband had a sister reported to have social communication difficulties, DLD, and, as we reported before, a 3.5 Mb deletion of Xp22.31–p22.33. This deletion was absent in the proband (Kalnak et al. 2018).

We identified a de novo variant in *TNRC6B* [NM_001162501.2(TNRC6B):c.830_836del (p.Asn277MetfsTer3)] in the proband F35.1 (Fig. 1D). Heterozygous mutations in *TNRC6B* cause a syndrome of global developmental delay with speech and behavioral abnormalities (OMIM 619243) (Eising et al. 2019). Speech delay was documented in 94% of individuals with pathogenic *TNRC6B* variants, followed by other forms of developmental delay, intellectual disability, ASD, ADHD, behavioral abnormalities, musculoskeletal abnormalities, and dysmorphic features (Granadillo et al. 2020).

### Variants in novel genes and genes with nonconclusive associations with DLD/NDD

**Fig. 2:**
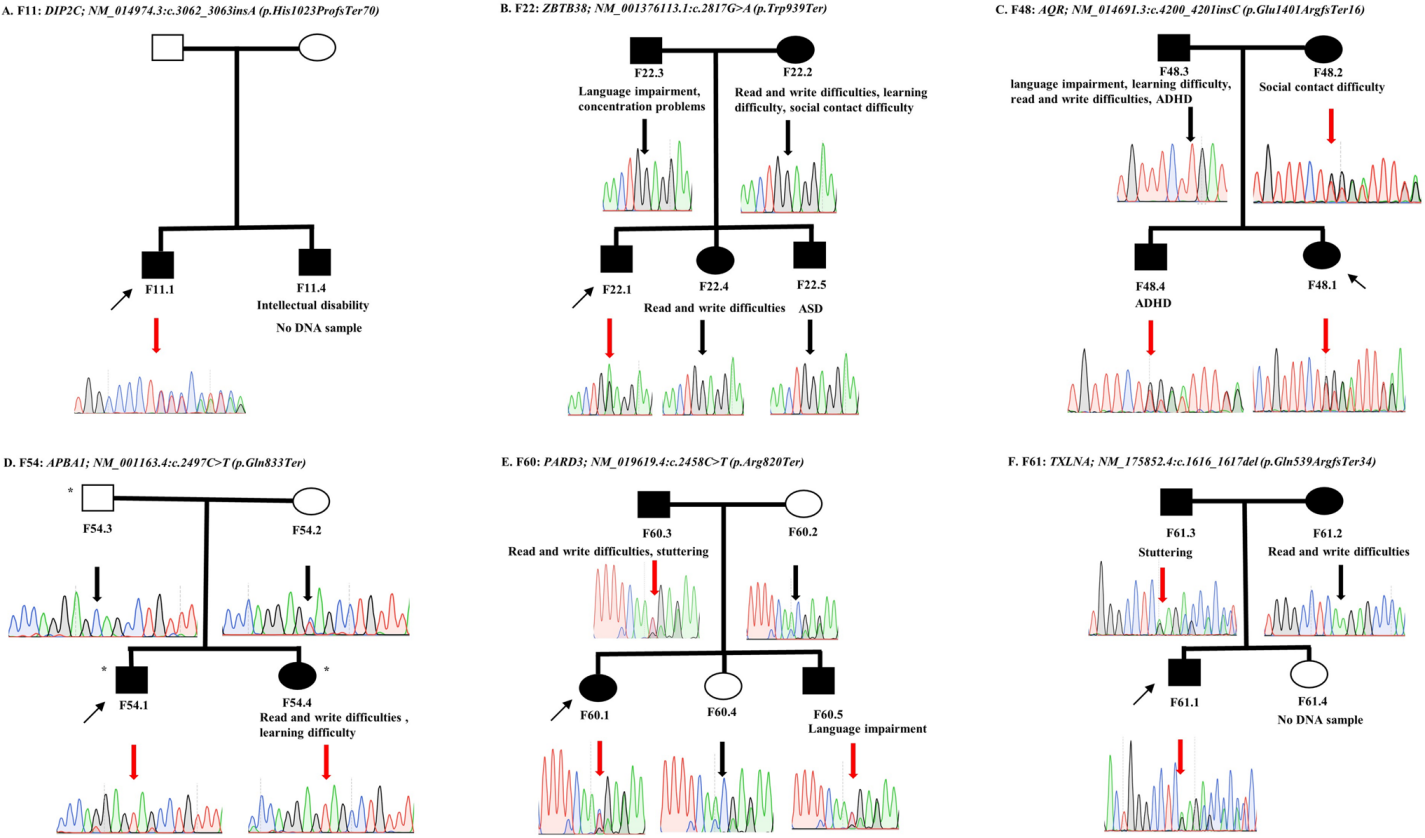
Segregation analysis of the variants identified in candidate novel genes and genes with no strong evidence that they cause neurodevelopmental disorders. **A**–**F** Thin arrows point to the proband in each family, thick red arrows point to culprit variants, black arrows point to wild-type variants, circles depict females, squares depict males, filled circles and squares depict affected individuals, and unfilled circles and squares depict controls. DLD, developmental language disorders; ADHD, attention-deficit hyperactivity disorder; ASD, autism spectrum disorder. * Individuals with a heterozygous deletion including *APBA1* exons 12 and 13

We identified four high-impact variants in four candidate novel genes (*ZBTB38*, *AQR*, *APBA1*, and *TXLNA*) not implicated before in NDD or genetic diseases in four probands/families (Table 2; Fig. 2B–D, F). Two of those variants were dominantly inherited from apparently affected parents, one variant was likely a de novo variant, and one, *APBA1*, was compound heterozygous with a paternal deletion (Fig. 2). The deletion in the father was identified in our earlier study showing a ~13 kb (chr9:72035199–72048477) heterozygous deletion including *APBA1* exons 12 and 13 and present in both siblings. We also identified two high-impact variants in two genes (*DIP2C* and *PARD3*) with yet nonconclusive associations with NDD (Table 2; Fig. 2A, E).

**Table 2:** The variants identified in genes with unknown/nonconclusive association with developmental language disorders or other neurodevelopmental disorders

Individual	Gene	Variant type	Variant	Protein size in amino acids^a^	Genotype/inheritance	gnomAD global frequency	pLI	O/E (90% CI)
F11.1	*DIP2C*	Frameshift insertion	NM_014974.3:c.3062_3063insA (p.His1023ProfsTer70)	1556	Heterozygous/unknown	Absent	1	0.08 (0.04–0.15)
F22.1	*ZBTB38*	Nonsense	NM_001376113.1:c.2817G>A (p.Trp939Ter)	1195	Heterozygous/de novo	Absent	1	0.09 (0.04–0.22)
F48.1	*AQR*	Frameshift insertion	NM_014691.3:c.4200_4201insC (p.Glu1401ArgfsTer16)	1485	Heterozygous/dominant	Absent	1	0.13 (0.08–0.22)
F54.1	*APBA1*	Nonsense	NM_001163.4:c.2497C>T (p.Gln833Ter)	837	Homozygous/unknown	Absent	0.54	0.21 (0.13–0.39)
F60.1	*PARD3*	Nonsense	NM_019619.4:c.2458C>T (p.Arg820Ter)	1356	Heterozygous/dominant	0.000004	0.96	0.2 (0.13–0.31)
F61.1	*TXLNA*	Frameshift deletion	NM_175852.4:c.1616_1617del (p.Gln539ArgfsTer34)	546	Heterozygous/dominant	Absent	1	0.08 (0.03–0.24)

O/E, observed/expected number of loss-of-function variants in gnomAD database; CI, confidence interval; pLI, probability of being loss-of-function intolerant according to gnomAD database

^a^Amino acid counts were according to RefSeq protein size

The pathogenicity of *DIP2C*, *TXLNA*, and *AQR* was supported by curating large datasets of genetic information of NDD and ASD individuals published by Zhou and colleagues (Zhou et al. 2022). Two loss-of-function *DIP2C* variants, NM_014974.3:c.2548del (p.Ser850ProfsTer10) and NM_014974.3:c.1969dup (p.Ala657GlyfsTer12), were reported in two ASD individuals out of 16,877 ASD individuals and 5,764 neurotypical controls (Zhou et al. 2022). Both variants were absent from the gnomAD v4.0 database. Similarly, one *TXLNA* frameshift variant, NM_175852.4:c.164del (p.Pro55ArgfsTer21), and one *AQR* nonsense variant, NM_014691.3:c.3136C>T (p.Arg1046Ter) (rs749141492), were each reported in one participant out of 31,058 with ASD (Zhou et al. 2022). NM_175852.4(TXLNA):c.164del (p.Pro55ArgfsTer21) was absent from gnomAD v4.0. On the other hand, rs749141492 was reported heterozygous in gnomAD v4.0 database with an allele frequency of 0.000002 in the European population.

### Differences in cognitive, speech, and language-related measures based on the genetic groups

We wanted to test if there were any differences in quantitative IQ and language-related measures between those with a putative genetic diagnosis (*n* = 16, also including the candidate genes) and those without (*n* = 43). We detected a statistically significant difference between these groups for NWR_PCC (*t* = 2.71, df = 59, two-tailed *p* value = 0.009). No statistically significant differences were detected in the other measures (Supplementary Table S1).

### Association with polygenic risk scores and the phenotypes

Next, we calculated PRS for two diagnoses (ASD and ADHD) and eight traits using earlier published source GWAS data (Demontis et al. 2023; Eising et al. 2022; Grove et al. 2019; Lee et al. 2018; Okbay et al. 2022). First, we tested if any significant differences could be seen in the distribution of the PRS values in the families by grouping the individuals, including probands, for neurodevelopmental difficulties (Supplementary Figs. S4–S13 for distribution of the PRS). Using GEE logistic regression models adjusted for the family structure, sex, and PC1–10, we did not detect any statistically significant association between neurodevelopmental difficulties and polygenic scores of ASD, ADHD, educational attainment, and cognitive performance (Supplementary Fig. S15).

Next, we used linear regression models to test available quantitative measures in the probands and the corresponding PRS values (Supplementary Table S2). No statistically significant associations were detected. Also, no significant differences were seen when we tested parents-to-probands PRS transmission disequilibrium for all the calculated PRS values (Supplementary Fig. S14).

## Discussion

Here, we studied the contribution of rare sequence-level variants to the genetic landscape of severe DLD in 53 individuals, followed by segregation analysis. Before, we have reported an aggregation of neurodevelopmental difficulties in the families of those probands (Kalnak et al. 2012, 2014). In a subsequent study, we reported, in the same cohort, an enrichment of rare CNVs in the probands but not in their family members (Kalnak et al. 2014). Herein, in our quest to explain the aggregation of these difficulties within those families, we investigated the association between the neurodevelopmental difficulties in the families and multiple neurodevelopmental traits/phenotypes PRS. We also investigated the association between multiple quantitative tests in the probands and their cognate PRS, and the transmission disequilibrium of neurodevelopmental traits/phenotypes PRS to the probands.

Our study adds to the growing evidence that individuals with DLD have similar rates of rare pathogenic genetic variants as in other NDD, ASD for instance (Srivastava et al. 2019). We also showed that individuals with monogenic/genomic DLD perform worse than those without monogenic/genomic DLD on a subtype of nonword repetition test, the percentage of correct consonants subtype. Nonword repetition has been identified as a clinical marker for Swedish-speaking school-aged children diagnosed with DLD (Kalnak et al. 2014) and in several other languages around the world (Schwob et al. 2021). A similar trend of scoring lower in many tests was seen but not significant. Therefore, further studies should investigate if indeed there are differences in these quantitative language measures within DLD based on the genetic subgrouping in larger samples.

We reached a molecular diagnostic yield of 7.5% (4/53) using WES. When we added on our earlier study for rare CNV contribution (Kalnak et al. 2018), the combined diagnostic yield in our cohort was 13.7% (8/58). A previous study reported a diagnostic yield of 26.8% on investigating a larger cohort of 127 DLD cases using a combination of karyotyping analysis, repeat expansion testing, targeted gene panels, SNP array genotyping, and WES (Plug et al. 2021). Our lower diagnostic yield was probably due to several reasons, including our smaller sample size, lesser range of genetic investigations used, and more conservative gene and variant pathogenicity cutoffs applied. In addition, we identified six variants in six different genes with less/no association with DLD/NDD in humans (Table 2; Fig. 2). The relevance of those candidate genes to DLD is discussed below.

*DIP2C* encodes a highly expressed protein in the brain and a member of the highly conserved DIP2 (Disco-interacting protein 2) family implicated in neuronal morphology and development and lipids’ metabolism (Ma et al. 2019; Mondal et al. 2022; Mukhopadhyay et al. 2002; Nitta et al. 2017; Noblett et al. 2019; Oo et al. 2020). A previous study reported 19 patients with submicroscopic deletion of chromosome 10p15.3 that included *DIP2C* and/or *ZMYND11* in all the cases. All the examined patients had developmental difficulties (*n* = 11) or language disorder (*n* = 10) (Descipio et al. 2012). A recent study reported a case of a 17-month-old female with focal infantile epilepsy, dysmorphic features, and developmental delays in motor developmental coordination and in receptive and expressive language (Yang et al. 2022). WES identified the de novo variant NM_014974.3 (DIP2C): c.1057 + 2T>G that was shown by minigene transfection assay to lead to *DIP2C* alternative splicing and an 80 bp deletion in Exon 8 (Yang et al. 2022). Furthermore, we detected two loss-of-function *DIP2C* variants in two ASD individuals upon mining published genetic data from 16,877 ASD individuals (Zhou et al. 2022).

*PARD3* is essential for cell polarity and nervous system development (Chen et al. 2017a, b; Hirose et al. 2022). Two CNVs that involved only *PARD3* were reported before in association with ASD and neural tube defects. The first was a 139 kb deletion in a case presented with craniorachischisis, cleft lip and palate, and bilateral adrenal hypoplasia (Chen et al. 2013). The second was a de novo 209 kb duplication in a Turkish individual with ASD (Özaslan et al. 2021). Regarding rare sequence-level variants, a heterozygous frameshift variant, NM_019619.4(PARD3): c.1012dupG (p.Glu338GlyfsTer26), was identified in six patients from a Chinese family manifesting nonsyndromic isolated cleft palate. Ethmoid plate patterning defects observed in zebrafish supported the gene’s candidacy (Cui et al. 2022).

ZBTB38 is a ubiquitously expressed ZNF transcription factor that belongs to the POZ/BTB family (Costoya 2007; Sasai et al. 2005). In mice, heterozygous loss of *ZBTB38* decreased the expression of *Nanog* and *Sox2* and led to embryonic developmental failure and early embryonic lethality (Nishio et al. 2022). Heterozygous *SOX2* loss-of-function leads to anophthalmia/microphthalmia syndrome (OMIM 206900) that almost always includes NDD, sometimes without ocular abnormalities (Amlie-Wolf et al. 2022; Fantes et al. 2003). Multiple members of the POZ/BTB family are also associated with monogenic NDD, e.g., *ZBTB7A* (OMIM 619769) (Ohishi et al. 2020), *ZBTB11* (OMIM 618383) (Fattahi et al. 2018)*, ZBTB18* (OMIM 612337) (de Munnik et al. 2014), and ZBTB20 (OMIM 259050) (Cordeddu et al. 2014).

*AQR* encodes a retinoic acid-responsive DNA/RNA helicase that serves in homologous recombination repair and resolving R-loops (Sakasai et al. 2017; Sam et al. 1998; Sollier et al. 2014). Multiple proteins are involved in R-loop regulation, including DNA/RNA helicases and RNA degrading enzymes. Mutations in R-loops regulatory proteins are associated with multiple cancers and neurological disorders (Khan and Danckwardt 2022). One prominent example is *SETX* that encodes a DNA/RNA helicase and when mutated causes a recessive form of spinocerebellar ataxia (OMIM 606002) and a dominant form of juvenile amyotrophic lateral sclerosis (OMIM 602433) (Chen et al. 2004; Moreira et al. 2004). Interestingly, we detected one nonsense *AQR* variant in an NDD individual upon curating published genetic data from 31,058 NDD patients (Zhou et al. 2022).

*APBA1* encodes a member in a putative presynaptic organizer complex (CASK/APBA1/LIN-7) essential for synaptogenesis and synaptic transmission (Brouwer et al. 2019; Butz et al. 1998; Leonoudakis et al. 2004; Motodate et al. 2019). Mutations in *CASK* cause variable NDD phenotypes (OMIM 300422 and 300749), while mutations in *LIN7B* were proposed to cause ASD (Becker et al. 2020; Mizuno et al. 2015; Najm et al. 2008; Piluso et al. 2009). We predicted that the nonsense variant NM_001163.4:c.2497C>T (p.Gln833Ter) in the two siblings from the family F54 would not abolish the APBA1 protein expression by nonsense-mediated decay, given its predicted termination merely five amino acids prior to its canonical endpoint. Yet, these five amino acids reside within the highly conserved C-terminal tail of the protein; thus, their absence may perturb the tail-mediated regulatory mechanisms.

*TXLNA* encodes α-taxilin, a constituent of the centriolar subdistal appendage essential for centrosomal microtubules’ anchorage (Ma et al. 2022). Mutations in genes encoding components of the distal and subdistal centriolar appendages are implicated in ciliopathies and developmental defects, including brain defects (Ma et al. 2023). For example, homozygous mutations in *NIN* and *KIF2A*, encoding two components of the subdistal centriolar appendage, are linked to Sickler syndrome type 7 (OMIM 614851) and a syndrome of complex brain malformations (OMIM 615411), respectively (Dauber et al. 2012; Poirier et al. 2013). Knockout of *TXLNA* in human retinal pigment epithelial-1 and HeLa cell lines impairs microtubules dynamics and its knockdown alters the centrosomal localization of CEP170, another component of the subdistal centriolar appendage (Ma et al. 2022). Interestingly, we detected one carrier with frameshift *TXLNA* variant upon curating published genetic data from 31,058 ASD participants (Zhou et al. 2022).

More complex inheritance patterns and scenarios constitute a major challenge in NDD, including DLD and other genetic conditions. These scenarios include dual molecular diagnosis, oligogenic inheritance, and polygenic inheritance (Centanni et al. 2015; Dale et al. 2020; Posey et al. 2017). Dual molecular diagnosis is possible in family F60, in which we detected a high-impact variant in *PARD3* (Fig. 2E) and a variant of uncertain significance in *CHD3* (Supplementary Fig. S3). Pathogenic variants in *CHD3* show variable expressivity in Snijders Blok–Campeau syndrome (OMIM 618205), while *PARD3* was associated before with neural tube defects, cleft lip, cleft palate, ASD, and bilateral adrenal hypoplasia (Chen et al. 2013; Cui et al. 2022; Özaslan et al. 2021; van der Spek et al. 2022). The variant in *CHD3* was a predicted splice-site variant, and we did not functionally validate its effect. Thus, we could not conclude whether the mild phenotype in family F60 was a case of variable expressivity, an unknown disease-modifying genetic interaction between *CHD3* and *PARD3*, or other modifying factors. Another challenge that we want to highlight is the shared symptomatology and pathways between DLD and other NDD. However, this challenge is rather an opportunity to readdress the NDD nosology and phenotype-based classifications and to consider more gene- or pathway-oriented classifications. The emerging concept of NDD continuum is a major leap forward to reformulating NDD nosology (Morris-Rosendahl and Crocq 2020). Our results were in line with the NDD continuum concept as despite excluding individuals with ASD and/or ID, we still identified pathogenic/likely pathogenic variants in genes that can cause ASD and/or ID.

Regarding our PRS analyses, we did not detect any association between probands PRS and the assessed quantitative clinical measures. Additionally, PRS for ASD, ADHD, educational attainment, and cognitive performance were not associated with neurodevelopmental difficulties in the families. A negative association between educational attainment PRS and language delay has been reported before (Dale et al. 2020). The lack of association between ASD, ADHD, educational attainment, and cognitive performance polygenic scores and neurodevelopmental difficulties in the participant families could be due to one or more causes. These causes include a true lack of association, the small size of our cohort, the impact of environmental factors complicating investigating the association between those traits and PRS, the heterogeneity in our cohort as while all the probands had DLD their family members could have other forms of neurodevelopmental difficulties, and, except for educational attainment and cognitive performance, the relatively small size of the base GWAS. The small size of our cohort is not ideal for polygenic associations, and larger studies should be conducted to investigate better associations between PRS and phenotypes in DLD probands. Similarly, there is a pressing need for larger GWAS for the language diagnoses and even other NDD.

### Study limitations

Our sample size was small for investigating the polygenic factors in DLD. Another limitation was the lack of functional validation of both variant and gene pathogenicity. This limitation is not unique to our study and constitutes a challenge for studying DLD and other genetic conditions. Mitigating strategies that support and encourage functional studies include sharing novel candidate genes and variants to platforms such as GeneMatcher (Sobreira et al. 2015) and ClinVar and initiatives such as the DECIPHER project (Firth et al. 2009). One example is the NM_001377142.1(PLCB4):c.1898G>A (p.Arg633His) that has been functionally validated after repeated detection in auriculocondylar syndrome 2 (Kanai et al. 2022). Another example is when we used the datasets published by Zhou and colleagues (Zhou et al. 2022) to support the pathogenicity of *DIP2C*, *TXLNA*, and *AQR*. Also, we identified several matches in GeneMatcher for future studies. Functional studies also come with limitations in that the results cannot always be extrapolated to humans (Damianidou et al. 2022).

Another limitation was that we did not perform a physical clinical examination for participants and their families which could have added a broader phenotypic description and could have resolved possible report bias. Also, we performed WES only in the probands which reduced our chances of identifying families with more than one genetic cause and discouraged us from looking for small CNVs in WES data.

## Conclusion

Achieving a diagnostic success rate of 7.5%, we provide clear evidence of the usefulness of WES in searching for molecular diagnoses in children with DLD. We also provide preliminary results, indicating that individuals without monogenic/genomic DLD perform better than those with monogenic/genomic DLD on the percentage of correct consonants nonword repetition test, a finding to be verified in larger cohorts. We did not detect significant associations between language quantitative measures and language-related polygenic scores. Overall, more genetic studies focusing on DLD and related speech and language phenotypes should be prioritized, so that sample size would be comparable to the other more studied NDD, such as ASD and ADHD.

### Supplementary Information

Below is the link to the electronic supplementary material.

Supplementary file1 (DOCX 4741 KB)

## Data Availability

The data supporting the findings of this study (not including participants’ personal information) are available from the corresponding author upon reasonable request and subject to necessary clearances. Clinically relevant variants have been submitted to ClinVar database (submission SUB13751313).
