# Supplementary material for: Whole exome sequencing and polygenic assessment of a Swedish cohort with severe developmental language disorder
Source: Hum Genet. 2024 Feb 1;143(2):169–83. doi: 10.1007/s00439-023-02636-z (PMC10881898; doi:10.1007/s00439-023-02636-z)
Supplement: Supplementary file 1 — Supplementary file1 (DOCX 4741 KB) [file 439_2023_2636_MOESM1_ESM.docx]

**Article title: Whole exome sequencing and polygenic assessment of a Swedish cohort with severe developmental language disorder**

**Journal Name: Human Genetics**

**Document: Supplementary material**

**Authors**

Ashraf Yahia^1,2^, Danyang Li^1,2,3^, Sanna Lejerkrans^1,2^, Shyam Rajagopalan^1,2,4^, Nelli Kalnak^1,5^ and Kristiina Tammimies^1,2^

**Affiliations**

^1^Center of Neurodevelopmental Disorders (KIND), Centre for Psychiatry Research, Department of Women's and Children's Health, Karolinska Institutet

^2^Astrid Lindgren Children's Hospital, Karolinska University Hospital, Region Stockholm, Stockholm, Sweden

^3^Social, Genetic and Developmental Psychiatry Centre, King's College London, London, the United Kingdom

^4^Institute of Bioinformatics and Applied Biotechnology, Bengaluru, India

^5^Department of Speech-Language Pathology, Helsingborg Hospital, Helsingborg, Sweden

**Correspondence:** Dr. Kristiina Tammimies ([kristiina.tammimies@ki.se](mailto:kristiina.tammimies@ki.se)).

**Variant of uncertain significance in *CHD3* in family F60**

In family F60, the proband carried the variant NM_001005271.3:c.2327A>G (p.Asp776Gly) in a heterozygous state affecting the *CHD3* gene (Fig.S2). The variant was inherited from the father, who had childhood literacy issues and a persisting stutter. The proband had a sister (individual F60.4) who also carried the variant. According to the parents, individual F60.4 was screened for language disorder and NDD and was not found to have any. The variant was absent in their brother (F60.5), who was also reported to have DLD. NM_001005271.3:c.2327A>G was predicted as a splice-site variant by TraP score (0.94) (Gelfman et al., 2017) and as a splice-donor variant by SpliceAI (0.91) (Jaganathan et al., 2019). However, AutoPVS1 did not support it as a PVS1 variant according to Abou Tayoun et al., 2018 recommendation. We classified NM_001005271.3:c.2327A>G as a variant of uncertain significance as it had a REVEL score of 0.726 (PP3_supporting according to Pejaver et al. 2022 recommendation), was absent from gnomAD v2.1 database (PM2), and did not segregate perfectly with the disease in family F60 (BS4). However, this classification can be changed in the future if additional functional or clinical evidence emerges. Heterozygous mutations in *CHD3* cause Snijders Blok-Campeau syndrome, characterized by developmental delay, speech delay, intellectual disability, and dysmorphic features (Drivas et al., 2020; Eising et al., 2019). Inherited variants in *CHD3* show variable expressivity in Snijders Blok-Campeau syndrome and are mostly maternally inherited (van der Spek et al., 2022).

**Supplementary figures**


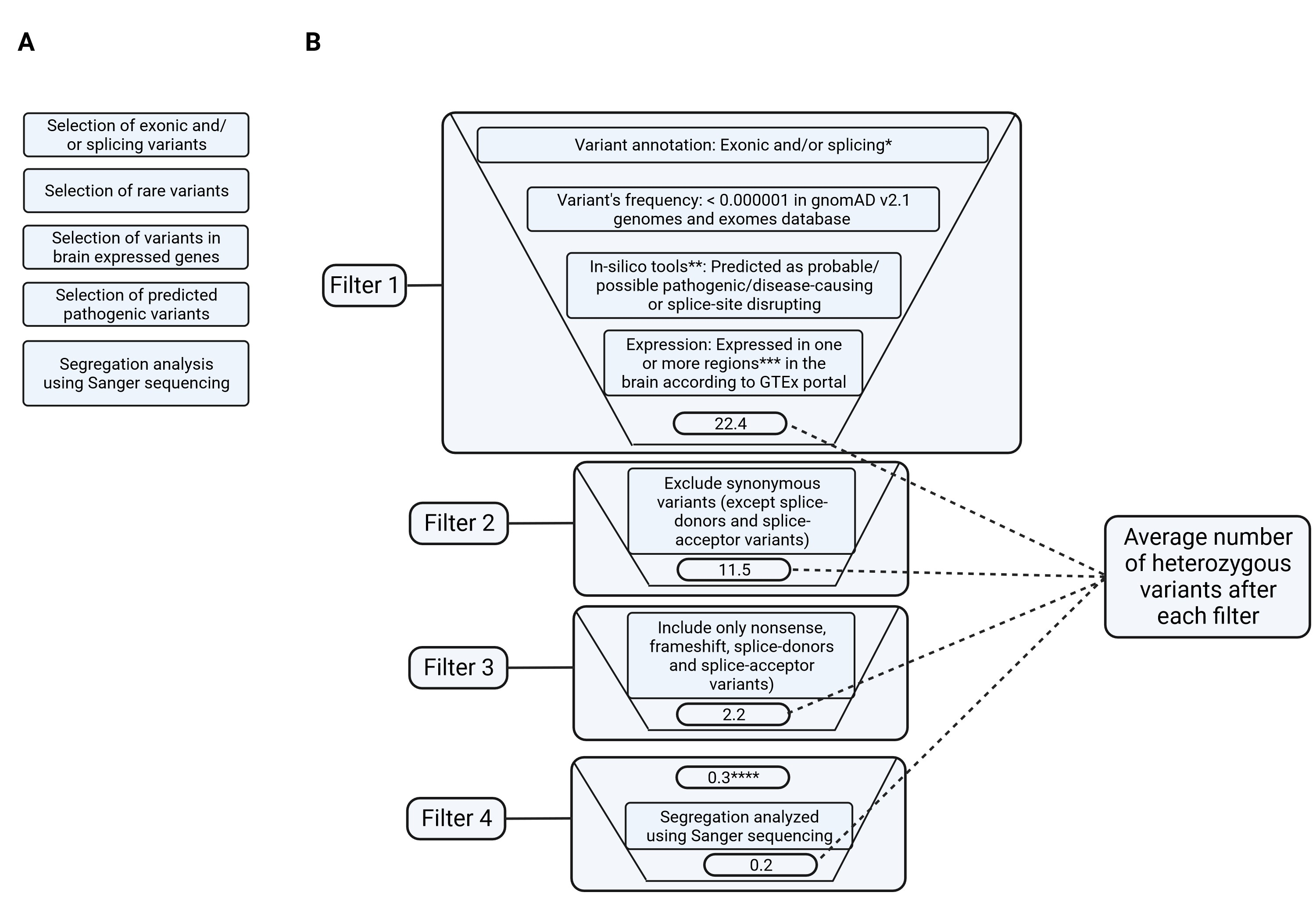


**Fig.S1. Variant filter thresholds**. A. A simplified overview of our filtration strategy. B. The filters used for the dominant/de novo model and the average number of variants that remained after each filter (calculated as the number of variants/53). * We only included splice-site variants at two nucleotides upstream or downstream to the transcription start site as variants at those locations have higher probability to be splice donor/acceptors variants. We used TraP scores > 0.676 (Gelfman et al., 2017) and SpliceAI delta scores > 8 (0.91) (Jaganathan et al., 2019) as cutoffs for splice-site variants ** Variants reported in ClinVar as likely pathogenic/pathogenic and match the disease inheritance pattern were directly sent for Snger sequencing and segregation analysis. SpliceAI and TraP scores were used to predict splice-site variants while SIFT and Polyphen were used for missense variants. Herein we are only reporting nonsense, splice-site, frameshift variants, or variants reported in ClinVar as pathogenic/likely pathogenic *** Expression is > 1 transcript per million (TPM) in one or more of the brain regions included in GTEx portal **** Only we included variants having genotypes that matches the genotype necessary/predicted necessary to develop a disease. We used gnomAD probability of being loss of function intolerant (pLI) > 0.9 as a cutoff to predict dominant genes.


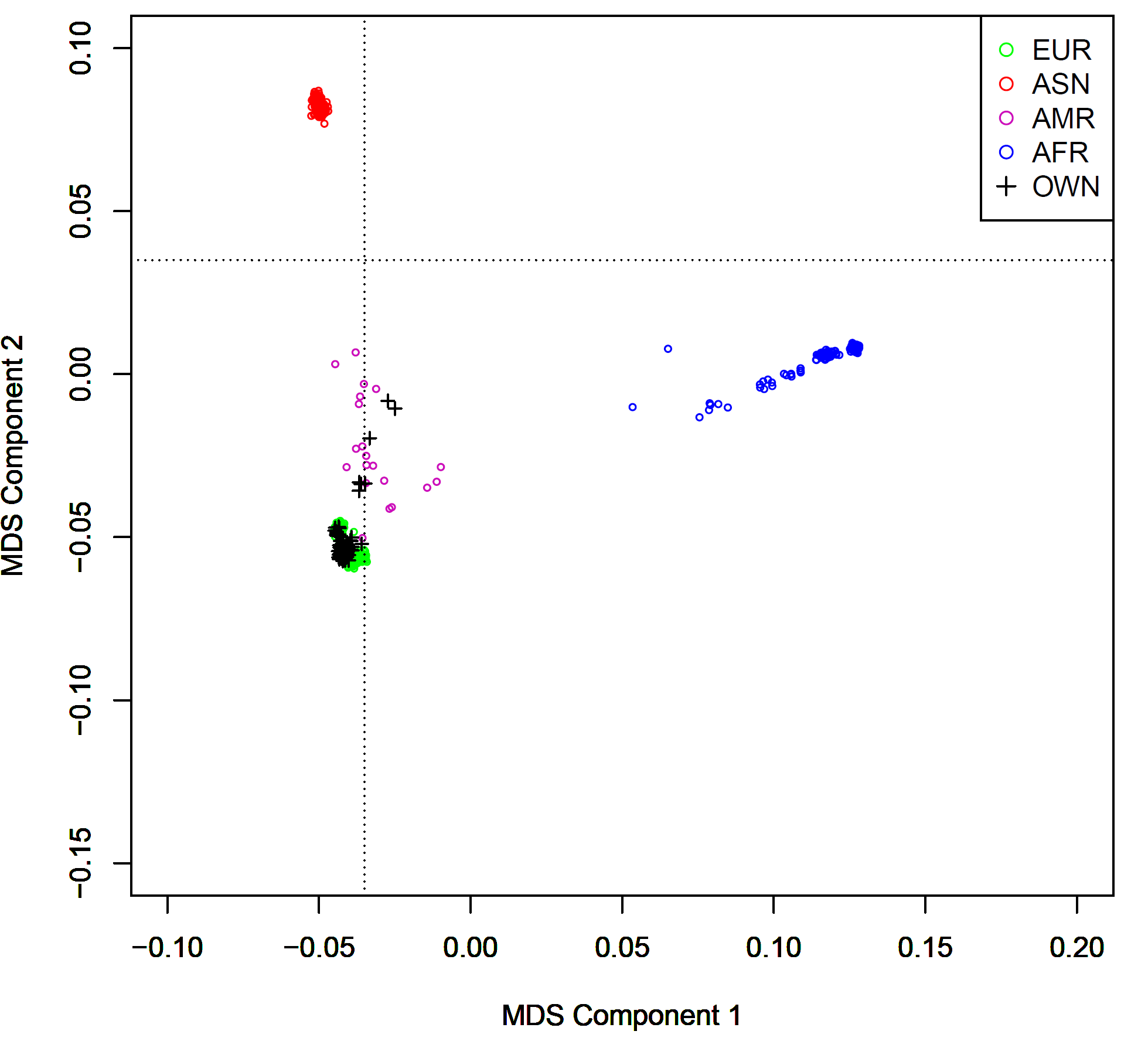


**Fig.S2. Multidimensional scaling (MDS) plot of our DLD dataset against phase 3 of the 1000 genome project data.** The black crosses represent the first two MDS components of the individuals in our dataset. The colored symbols represent phase 3 of the 1000 genome project data. MDS, multidimensional scaling; EUR, European; AFR, African; AMR, Mixed American; ASN, Asian.


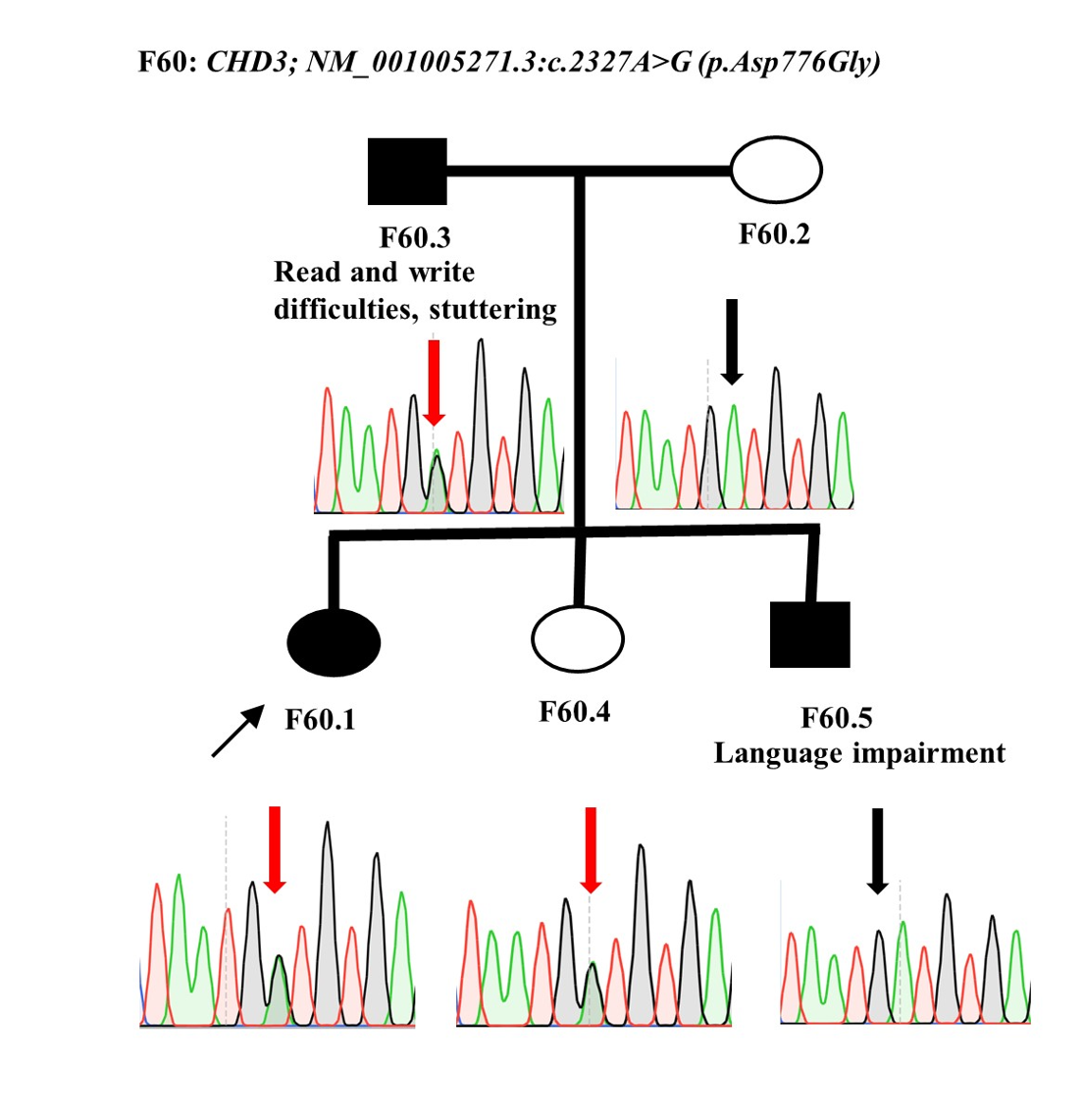


**Fig.S3. Segregation analysis** of the variants NM_001005271.3:c.2327A>G (p.Asp776Gly) identified in family F60. Thin arrow points to the proband, thick red arrows point to the variant, and black arrows point to wild-type variant, circles depict females, squares depict males, filled circles and squares depict patients, unfilled circles and squares depict controls.


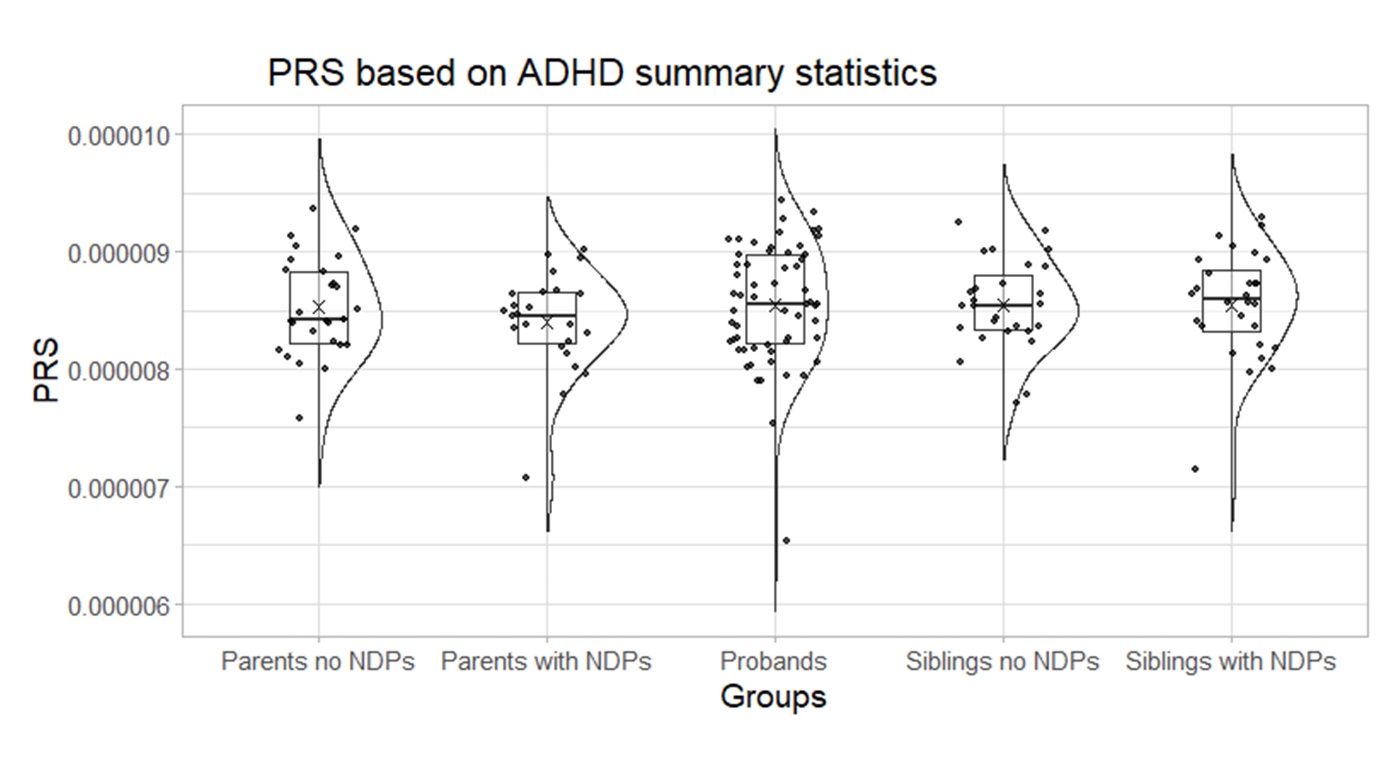


**Fig.S4. PRS based on ADHD summary statistics**. Demontis et al., 2023, was used as a base data source to calculate PRS. No significant difference was noted between the different groups. ADHD, Attention-deficit hyperactivity disorder; PRS, Polygenic Risk Score; NDPs, neurodevelopmental phenotypes.


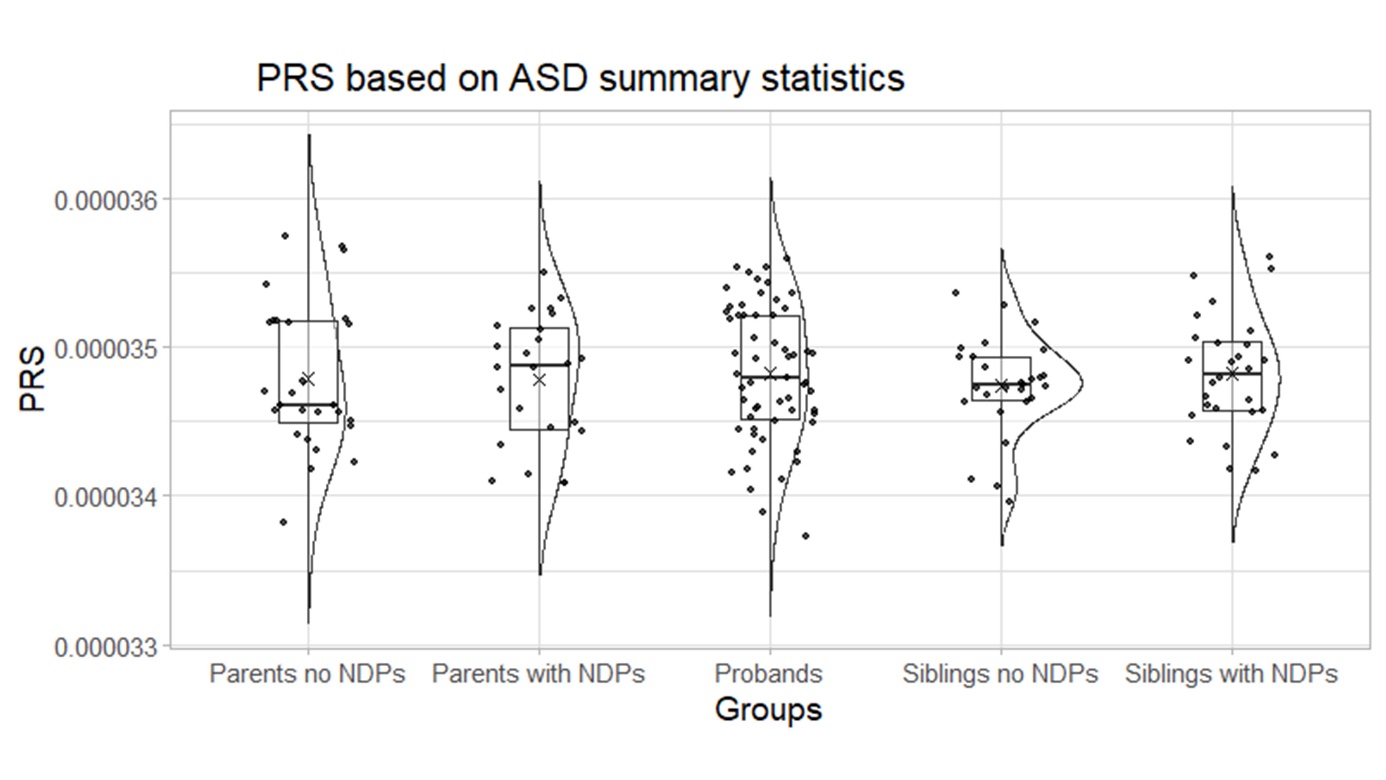


**Fig.S5. PRS based on ASD summary statistics**. Grove et al., 2019, was used as a base data source to calculate PRS. No significant difference was noted between the different groups. ASD, Autism spectrum disorder; PRS, Polygenic Risk Score; NDPs, neurodevelopmental phenotypes.


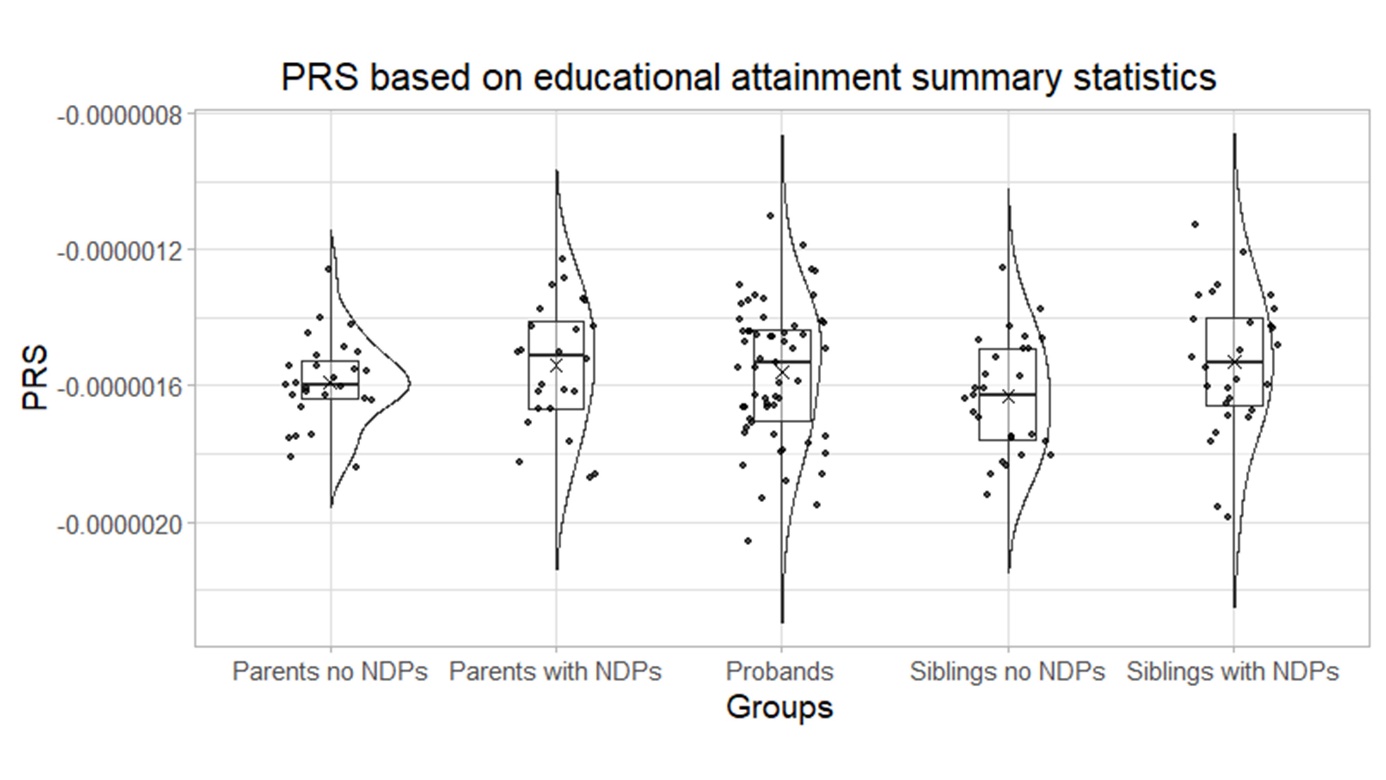


**Fig.S6. PRS based on educational attainment summary statistics**. Okbay et al., 2022, was used as a base data source to calculate PRS. No significant difference was noted between the different groups. PRS, Polygenic Risk Score; NDPs, neurodevelopmental phenotypes.


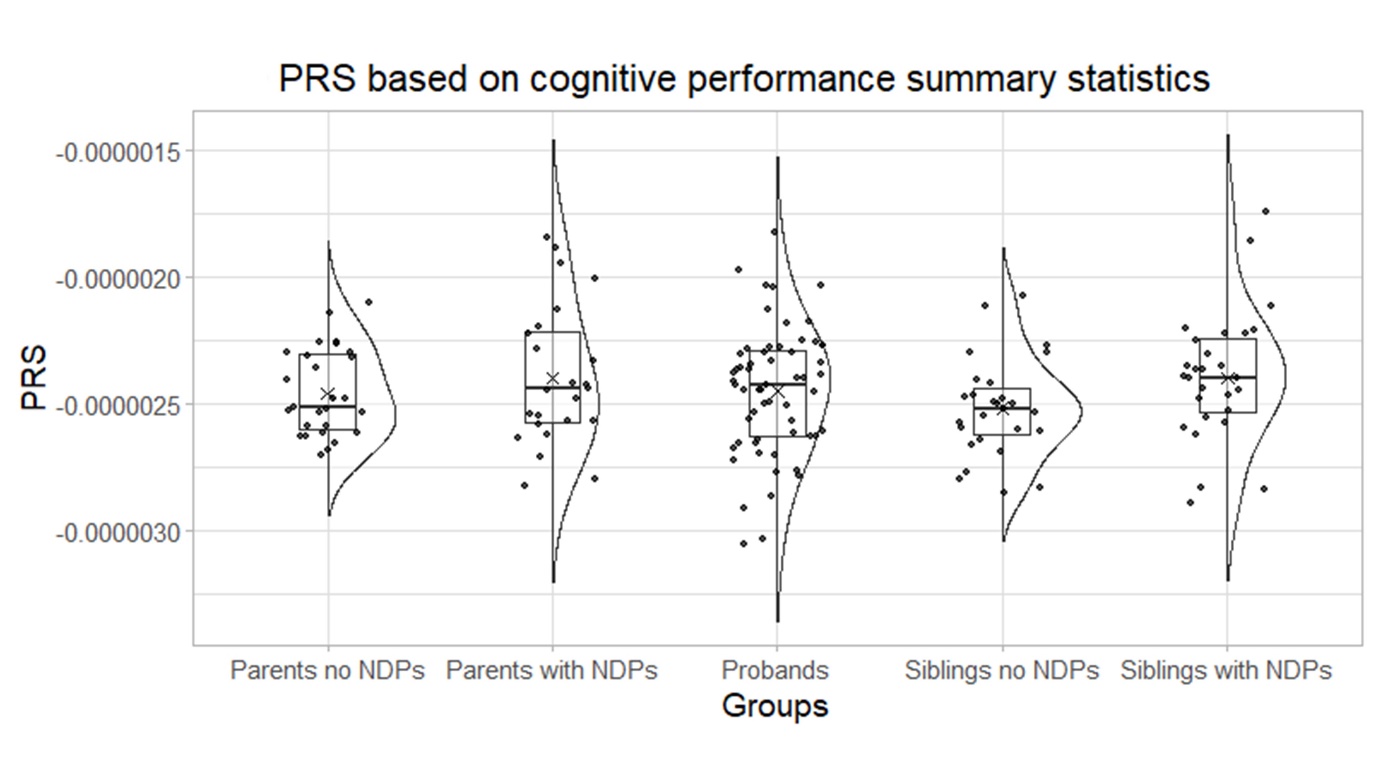


**Fig.S7. PRS based on cognitive performance summary statistics.** Lee et al., 2018, was used as a base data source to calculate PRS. No significant difference was noted between the different groups. PRS, Polygenic Risk Score; NDPs, neurodevelopmental phenotypes.


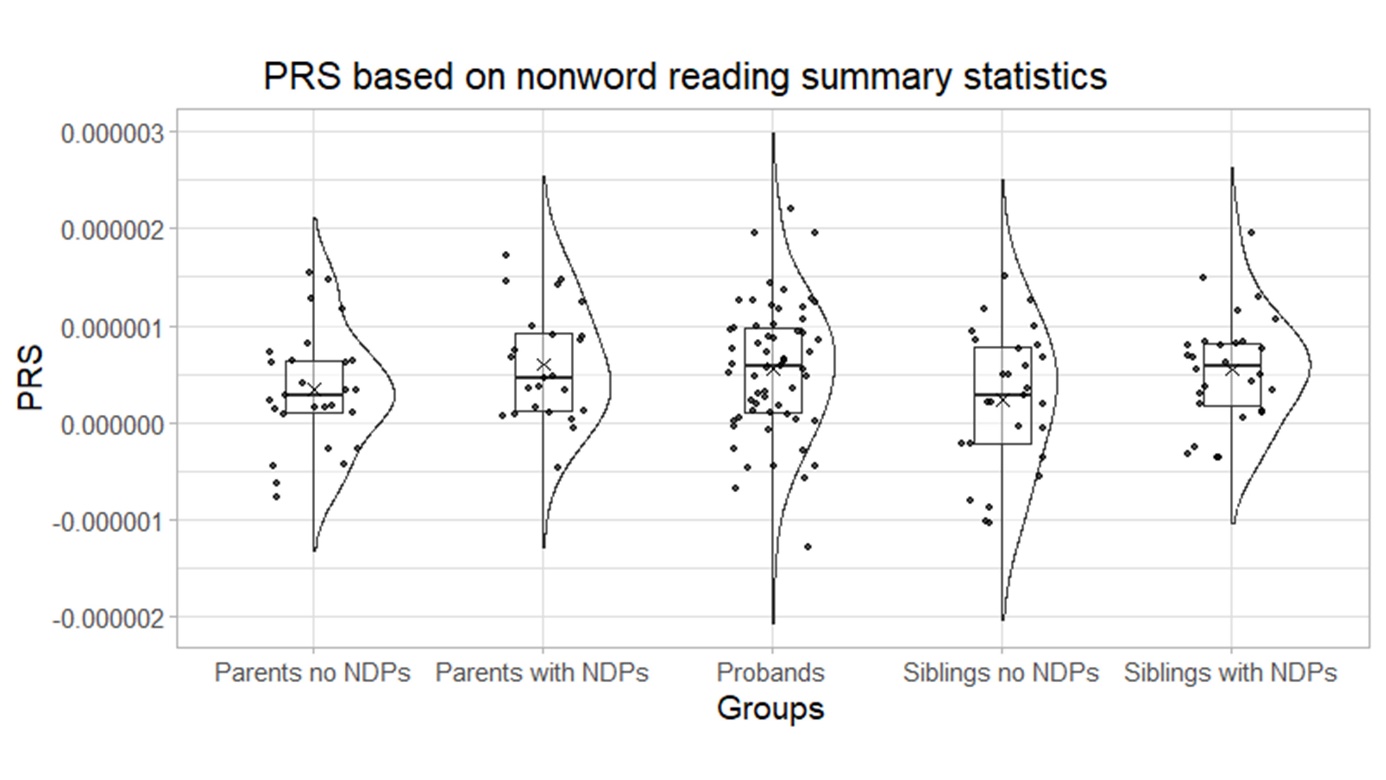


**Fig.S8. PRS based on nonword reading summary statistics**. Eising et al., 2022, was used as a base data source to calculate PRS. No significant difference was noted between the different groups. PRS, Polygenic Risk Score; NDPs, neurodevelopmental phenotypes.


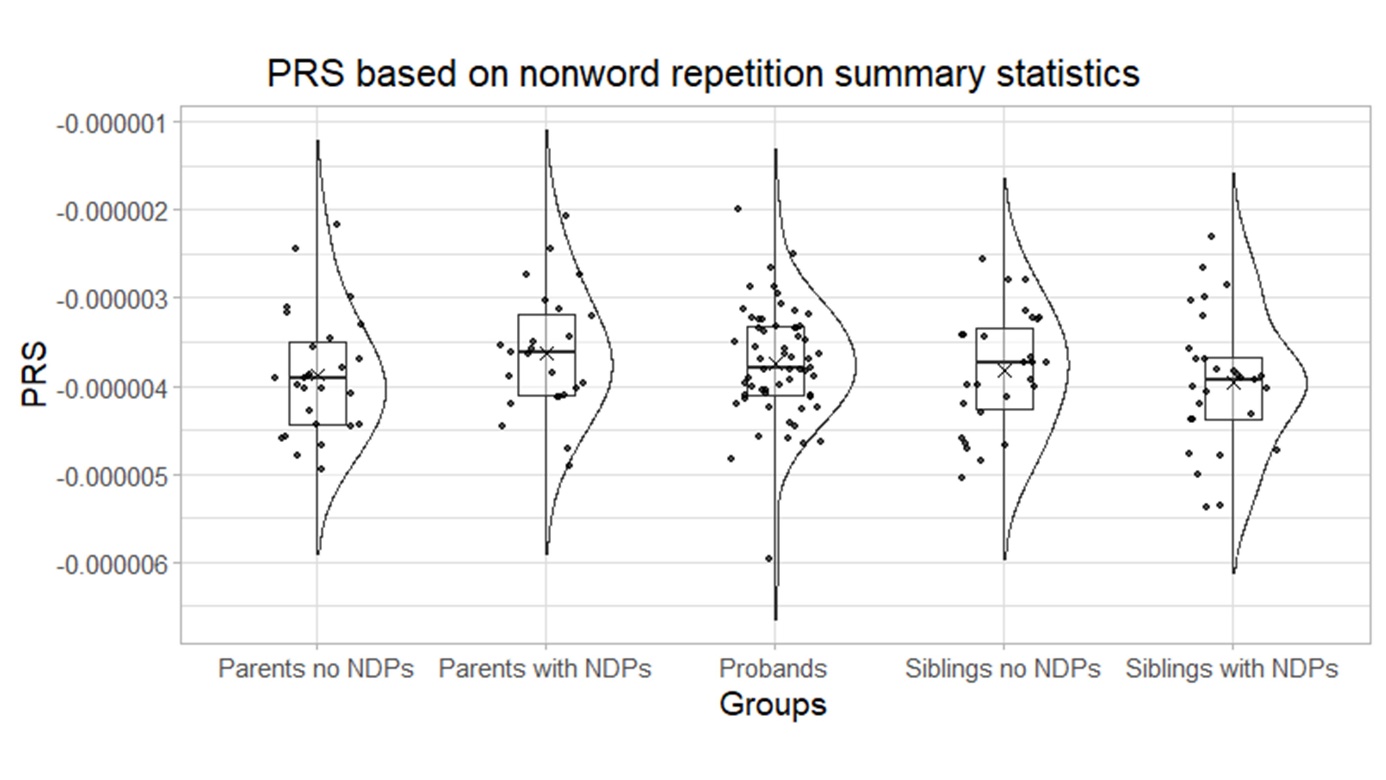


**Fig.S9. PRS based on nonword repetition summary statistics**. Eising et al., 2022, was used as a base data source to calculate PRS. No significant difference was noted between the different groups. PRS, Polygenic Risk Score; NDPs, neurodevelopmental phenotypes.


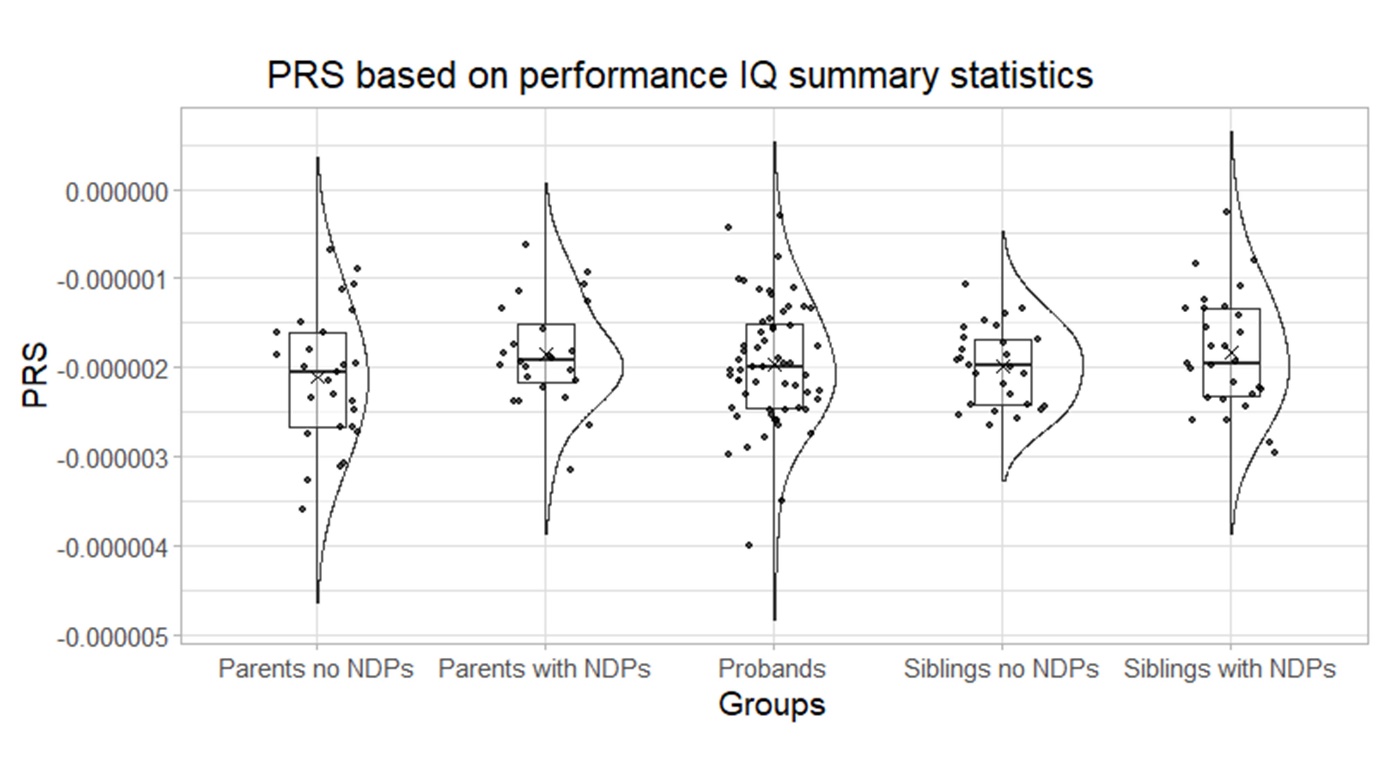


**Fig.S10. PRS based on performance IQ summary statistics**. Eising et al., 2022, was used as a base data source to calculate PRS. No significant difference was noted between the different groups. PRS, Polygenic Risk Score; NDPs, neurodevelopmental phenotypes.


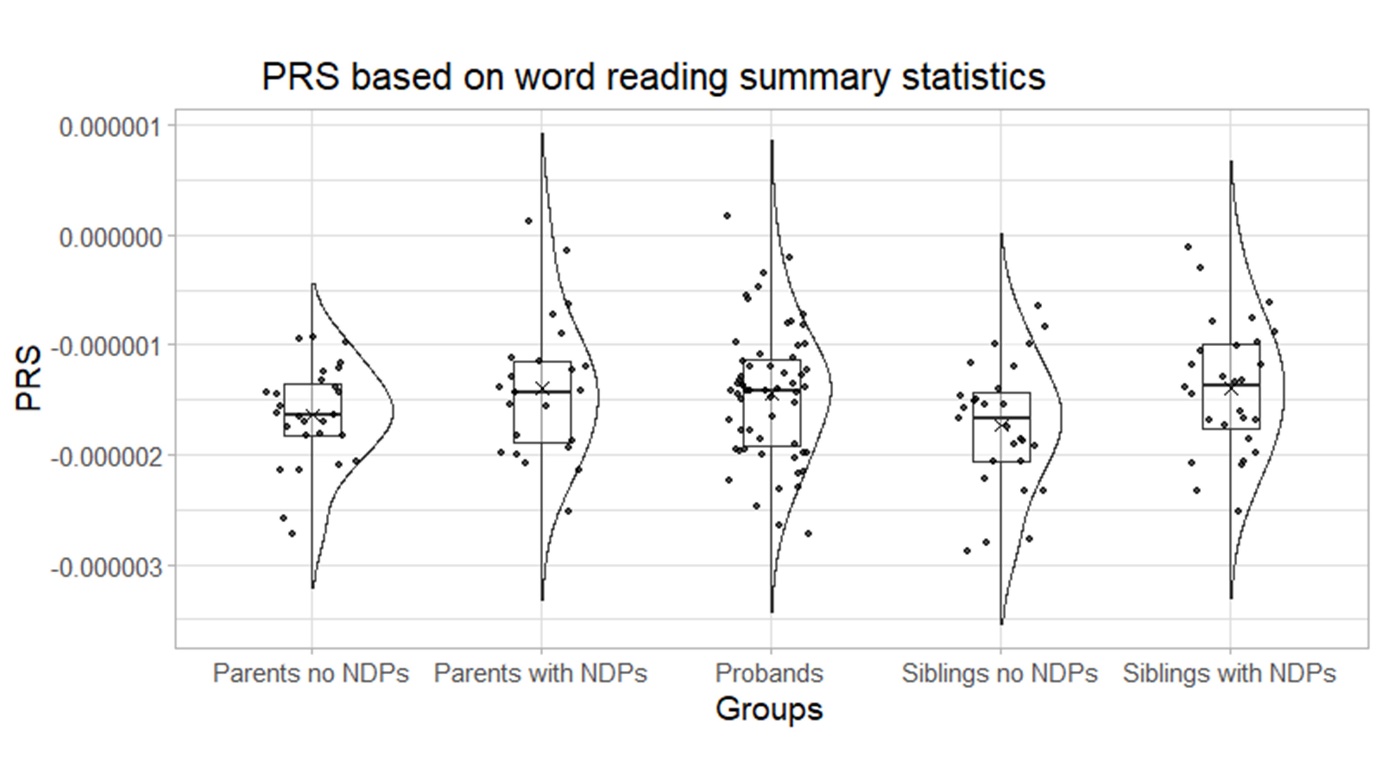


**Fig.S11. PRS based on word reading summary statistics**. Eising et al., 2022, was used as a base data source to calculate PRS. No significant difference was noted between the different groups. PRS, Polygenic Risk Score; NDPs, neurodevelopmental phenotypes.


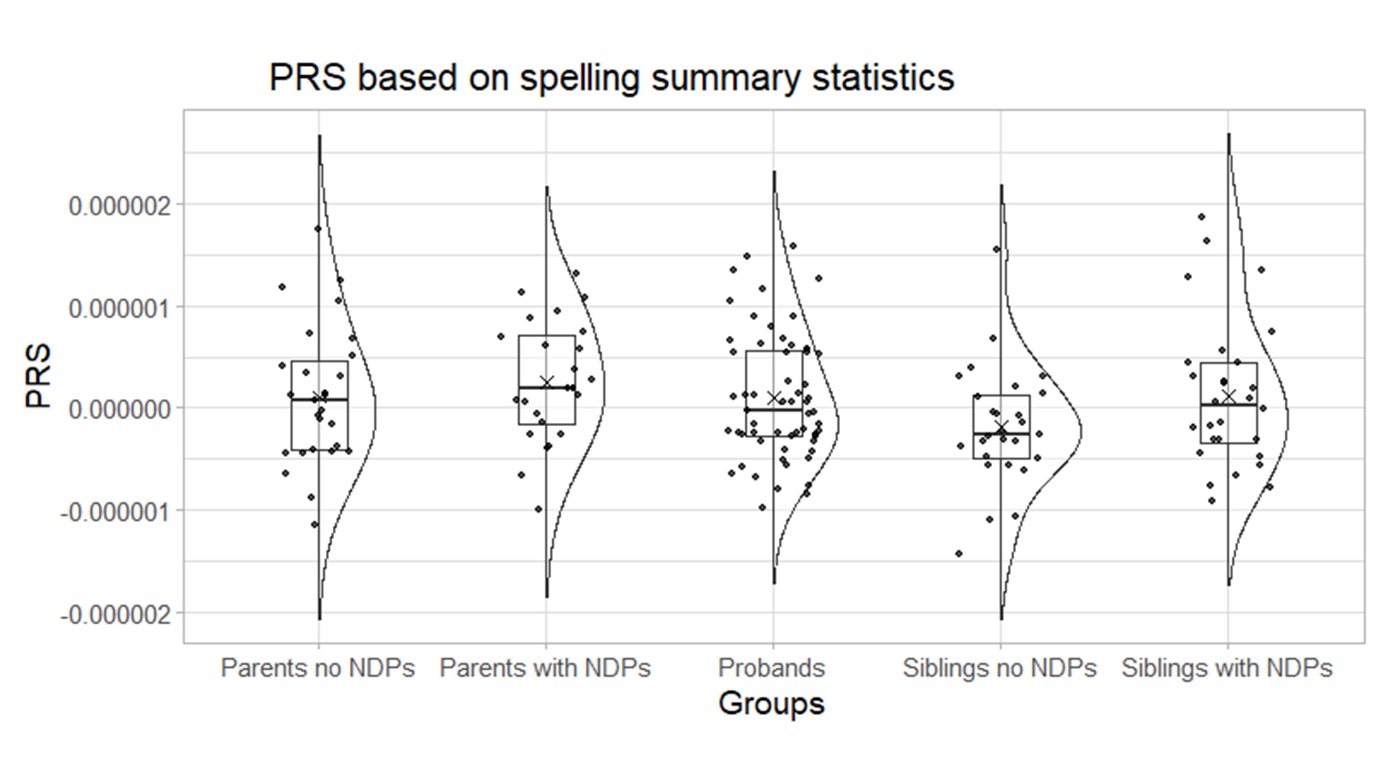


**Fig.S12. PRS based on spelling summary statistics**. Eising et al., 2022, was used as a base data source to calculate PRS. No significant difference was noted between the different groups. PRS, Polygenic Risk Score; NDPs, neurodevelopmental phenotypes.


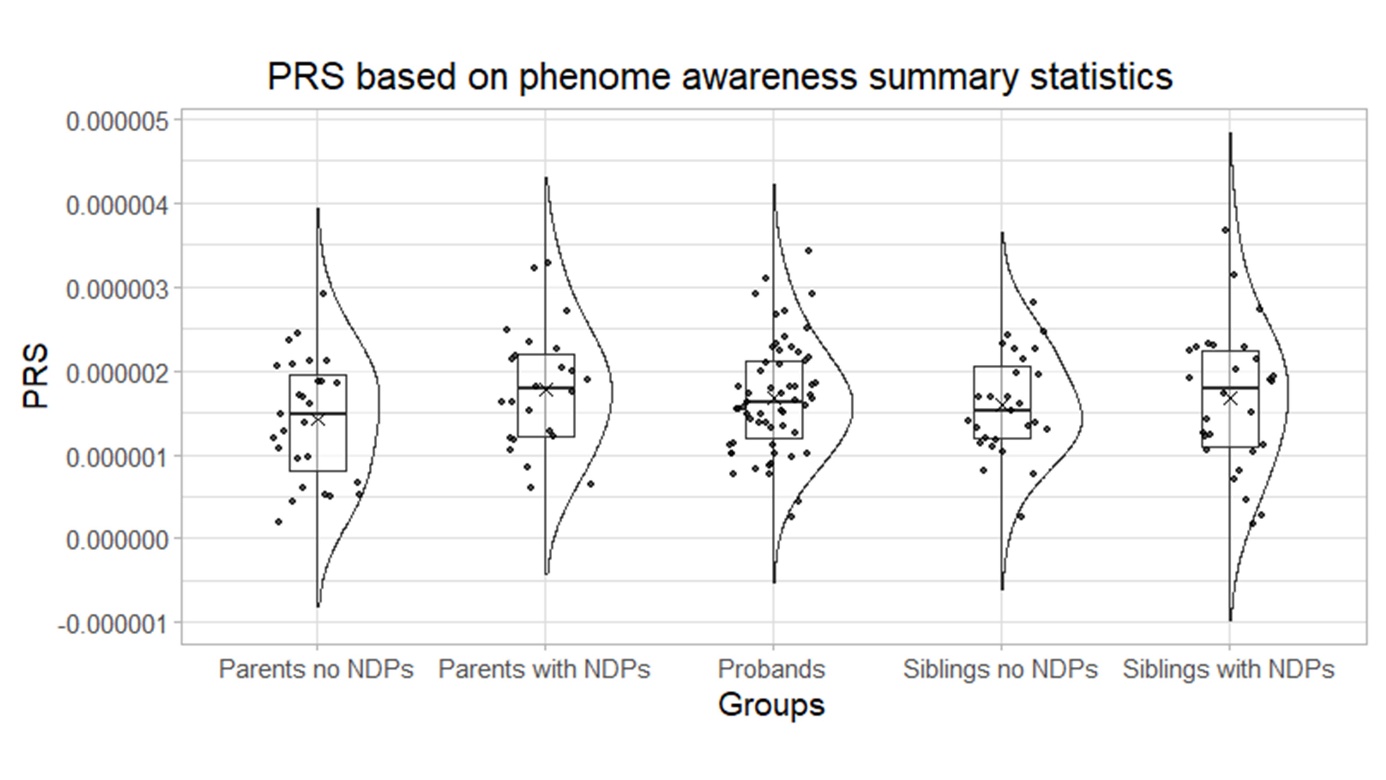


**Fig.S13. PRS based on phenome awareness summary statistics**. Eising et al., 2022, was used as a base data source to calculate PRS. No significant difference was noted between the different groups. PRS, Polygenic Risk Score; NDPs, neurodevelopmental phenotypes.


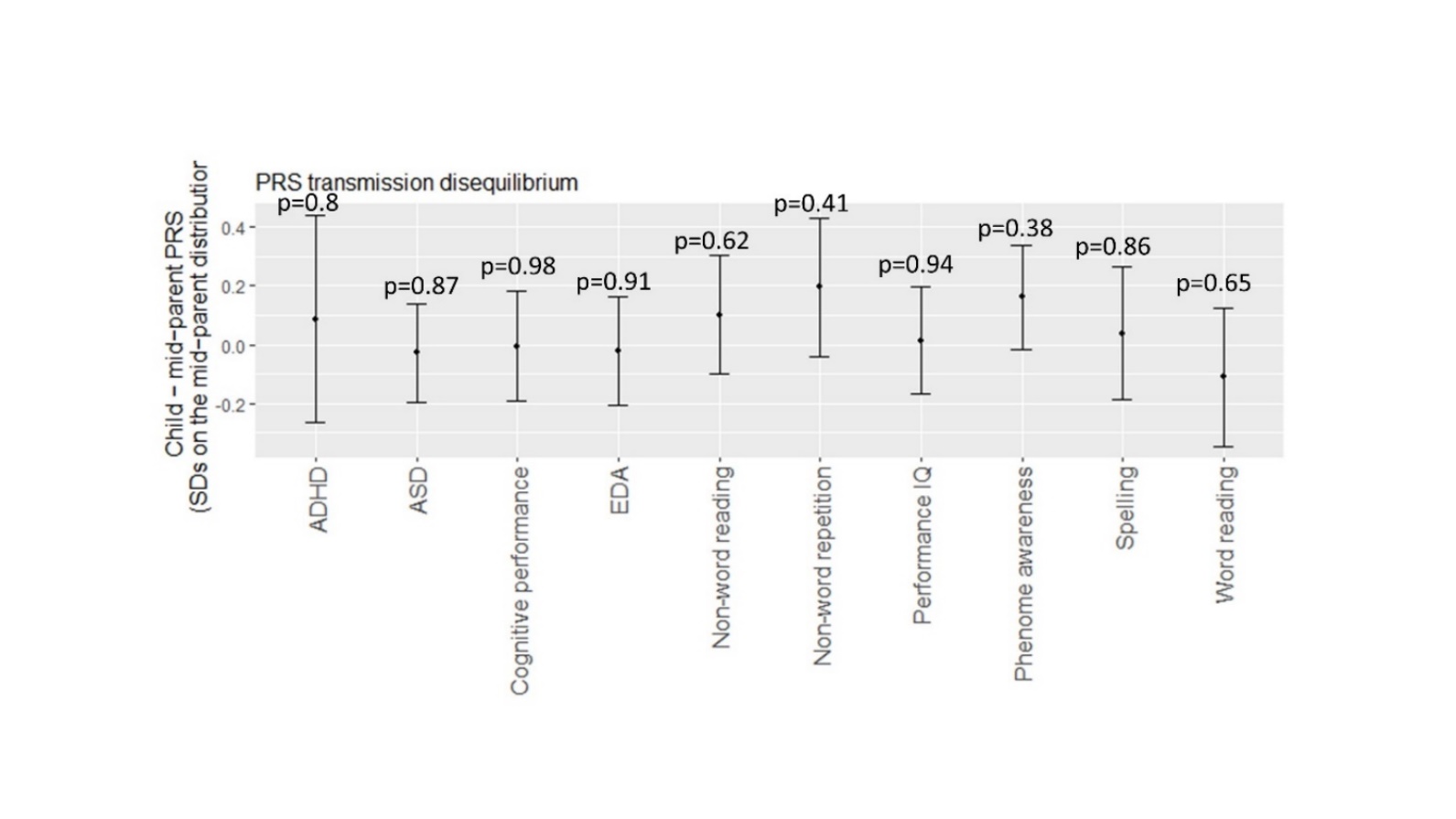


**Fig.S14. Parents to probands PRS transmission disequilibrium test**. PRS transmission disequilibrium was tested in 30 patients from 20 families using ptdt tool generated by Weiner et al., 2017. Transmission disequilibrium is shown in terms of standard deviation on the mid-parent distribution ± 1.96 standard error (95% confidence intervals). P values indicate the probability that the mean of the pTDT deviation distribution is 0 (two-sided, one-sample t test). No statistically significant over-inheritance of the assessed PRS was noted. ADHD, Attention-deficit hyperactivity disorder; ASD, Autism spectrum disorder; EDA, educational attainment; PRS, polygenic risk score; SDs, standard deviations.


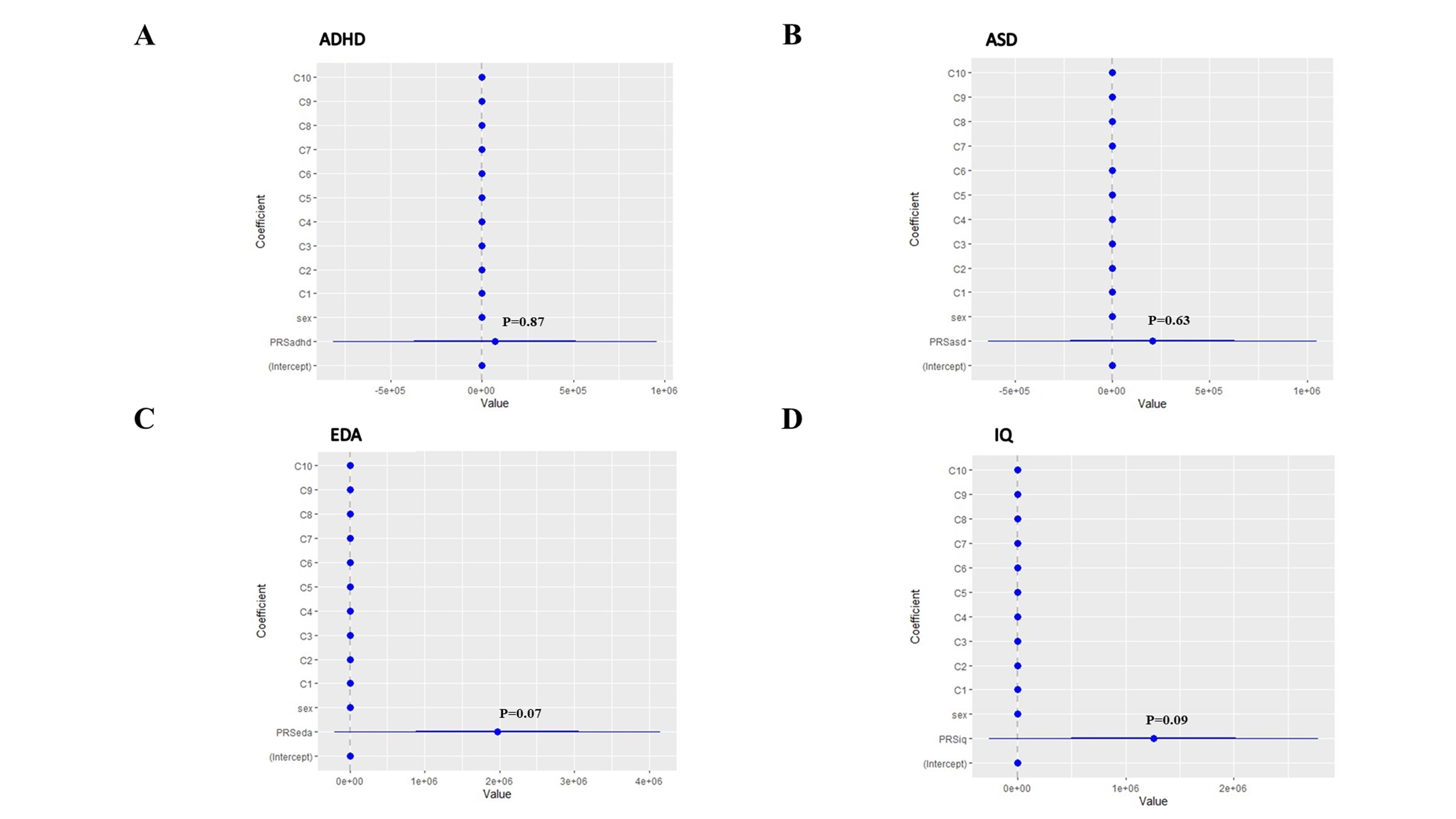


**Fig.S15.** Generalized estimating equations (GEE) model to test the association between DLD (or other neurodevelopmental phenotypes) and polygenic scores for attention-deficit hyperactivity disorder (A), autism spectrum disorder (B), educational attainment (C), and cognitive performance (D) adjusted for family structure, sex, and the first ten principal components. Educational attainment and IQ polygenic risk scores showed a statistically significant but negligible association. No other statistically significant associations were detected. ADHD, attention-deficit hyperactivity disorder; ASD, autism spectrum disorder; EDA, educational attainment; PRS, polygenic risk scores; C1-10, the first ten principal components.

**Supplementary tables**

**Table S1.** Statistical analysis of the performance of the probands with a likely monogenic diagnosis vs those without a monogenic diagnosis on Raven’s colored progressive matrices (CPM9), non-word repetition task (NWR), and test of word-reading efficiency (TOWRE) tests. CPM9, NWR, and TOWRE were expressed in standard deviation units (z-scores). NWR z-scores were calculated based on whole-word accuracy (binary outcome of either a positive or negative response; NWR_binary), numbers of syllabi (NWR_length), and percent of correct consonants (NWR_PCC). TOWRE z-scores were based on number of words (TOWRE_words) the number of non-words (TOWRE_nonwords). Unpaired student t-test detected a statistically significant difference in the percent of correct consonants during non-word reading (NWR_PCC) between the two groups.

| CPM9 | Description | | | |
| --- | --- | --- | --- | --- |
|  | Group | Number | Mean | Standard deviation |
|  | Group 1: Genomic/monogenic condition | 16 | 96.87 | 14 |
|  | Group 2: No high-impact genetic findings | 45 | 100.22 | 14.5 |
|  | Student t-test | | | |
|  | t-value | 0.80 | | |
|  | Degrees of freedom | 59 | | |
|  | Two-tailed p-value | 0.43 | | |
|  | Standard error of the difference | 4.19 | | |
|  | | | | |
| NWR_binary | Description | | | |
|  | Group | Number | Mean | Standard deviation |
|  | Group 1: Genomic/monogenic condition | 16 | -8.72 | 5.2 |
|  | Group 2: No high-impact genetic findings | 45 | -8.52 | 5.5 |
|  | Student t-test | | | |
|  | t-value | 0.13 | | |
|  | Degrees of freedom | 59 | | |
|  | Two-tailed p-value | 0.89 | | |
|  | Standard error of the difference | 1.58 | | |
|  | | | | |
| NWR_length | Description | | | |
|  | Group | Number | Mean | Standard deviation |
|  | Group 1: Genomic/monogenic condition | 16 | -7.8 | 9.92 |
|  | Group 2: No high-impact genetic findings | 45 | -4.74 | 6.09 |
|  | Student t-test | | | |
|  | t-value | 1.45 | | |
|  | Degrees of freedom | 59 | | |
|  | Two-tailed p-value | 0.15 | | |
|  | Standard error of the difference | 2.11 | | |
|  | | | | |
| NWR_PCC | Description | | | |
|  | Group | Number | Mean | Standard deviation |
|  | Group 1: Genomic/monogenic condition | 16 | -5.81 | 2.82 |
|  | Group 2: No high-impact genetic findings | 45 | -3.69 | 2.64 |
|  | Student t-test | | | |
|  | t-value | 2.71 | | |
|  | Degrees of freedom | 59 | | |
|  | Two-tailed p-value | 0.009 | | |
|  | Standard error of the difference | 0.78 | | |
|  | | | | |
| TOWRE_words | Description | | | |
|  | Group | Number | Mean | Standard deviation |
|  | Group 1: Genomic/monogenic condition | 16 | -2.01 | 1.17 |
|  | Group 2: No high-impact genetic findings | 45 | -1.77 | 1.36 |
|  | Student t-test | | | |
|  | t-value | 0.64 | | |
|  | Degrees of freedom | 59 | | |
|  | Two-tailed p-value | 0.53 | | |
|  | Standard error of the difference | 0.38 | | |
|  | | | | |
| TOWRE_nonwords | Description | | | |
|  | Group | Number | Mean | Standard deviation |
|  | Group 1: Genomic/monogenic condition | 16 | -2.22 | 0.89 |
|  | Group 2: No high-impact genetic findings | 45 | -1.88 | 1.35 |
|  | Student t-test | | | |
|  | t-value | 0.91 | | |
|  | Degrees of freedom | 59 | | |
|  | Two-tailed p-value | 0.37 | | |
|  | Standard error of the difference | 0.37 | | |

**Table S2.** Associations between six language-related clinical measures and their cognate polygenic risk scores using linear regression. Age, sex, the presence of a mongenic diagnosis, and first ten principal components (C1-C10) were added as covariables. The used clinical measures were CPM9, NWR, and TOWRE and were expressed in standard deviation units (z-scores). NWR z-scores were calculated based on whole-word accuracy (binary outcome of either a positive or negative response; NWR_binary), numbers of syllabi (NWR_length), and percent of correct consonants (NWR_PCC). TOWRE z-scores were based on number of words (TOWRE_words). NWR, non-word repetition task; CPM9, Raven’s colored progressive matrices; TOWRE, test of word-reading efficiency; C1-10, the first ten principal component analysis.

| **Linear regression model** | | **Estimate** | **Std. Error** | **t value** | **Pr(>\|t\|)** |
| --- | --- | --- | --- | --- | --- |
| CPM9 vs cognitive performance PRS | Intercept | 2.30E+02 | 1.37E+02 | 1.67 | 0.102 |
|  | Cognitive performance PRS | -1.01E+07 | 8.43E+06 | -1.2 | 0.239 |
|  | Age | -1.58E+00 | 1.96E+00 | -0.81 | 0.425 |
|  | Sex | 5.72E+00 | 4.51E+00 | 1.27 | 0.211 |
|  | Monogenic | -5.91E+00 | 4.97E+00 | -1.19 | 0.242 |
|  | C1 | 1.54E+04 | 1.10E+04 | 1.4 | 0.17 |
|  | C2 | 6.48E+02 | 3.02E+03 | 0.21 | 0.831 |
|  | C3 | -1.04E+02 | 1.08E+03 | -0.1 | 0.924 |
|  | C4 | -5.81E+01 | 1.81E+03 | -0.03 | 0.975 |
|  | C5 | -1.11E+03 | 5.06E+02 | -2.2 | 0.034 |
|  | C6 | 5.61E+02 | 5.10E+02 | 1.1 | 0.277 |
|  | C7 | -3.12E+02 | 6.79E+02 | -0.46 | 0.649 |
|  | C8 | -6.61E+02 | 4.91E+02 | -1.35 | 0.185 |
|  | C9 | -6.94E+01 | 5.94E+01 | -1.17 | 0.25 |
|  | C10 | 4.27E+01 | 3.92E+01 | 1.09 | 0.283 |
|  |  |  |  |  |  |
| CPM9 vs performance IQ PRS | Intercept | 2.81E+02 | 1.33E+02 | 2.11 | 0.04 |
|  | Performance IQ PRS | -6.66E+05 | 3.13E+06 | -0.21 | 0.833 |
|  | Age | -2.12E+00 | 1.95E+00 | -1.09 | 0.282 |
|  | Sex | 5.86E+00 | 4.58E+00 | 1.28 | 0.207 |
|  | Monogenic | -5.16E+00 | 5.37E+00 | -0.96 | 0.342 |
|  | C1 | 1.79E+04 | 1.10E+04 | 1.62 | 0.112 |
|  | C2 | 5.46E+02 | 3.07E+03 | 0.18 | 0.86 |
|  | C3 | 1.34E+02 | 1.08E+03 | 0.12 | 0.902 |
|  | C4 | -4.10E+02 | 1.83E+03 | -0.22 | 0.824 |
|  | C5 | -1.22E+03 | 5.13E+02 | -2.38 | 0.022 |
|  | C6 | 6.84E+02 | 5.12E+02 | 1.34 | 0.189 |
|  | C7 | -4.67E+02 | 6.77E+02 | -0.69 | 0.494 |
|  | C8 | -6.67E+02 | 4.99E+02 | -1.34 | 0.189 |
|  | C9 | -7.45E+01 | 6.03E+01 | -1.24 | 0.223 |
|  | C10 | 4.23E+01 | 4.00E+01 | 1.06 | 0.297 |
|  |  |  |  |  |  |
| NWR_binary vs non-wordrepetition PRS | Intercept | -3.64E+01 | 4.42E+01 | -0.83 | 0.41 |
|  | Non-word repetition PRS | 2.53E+05 | 9.87E+05 | 0.26 | 0.8 |
|  | Age | 3.22E+00 | 6.46E-01 | 4.98 | 1.10E-05 |
|  | Sex | -1.41E+00 | 1.52E+00 | -0.93 | 0.36 |
|  | Monogenic | -1.54E+00 | 1.65E+00 | -0.93 | 0.36 |
|  | C1 | 1.17E+03 | 3.68E+03 | 0.32 | 0.75 |
|  | C2 | 7.55E+02 | 1.02E+03 | 0.74 | 0.46 |
|  | C3 | -1.06E+02 | 3.58E+02 | -0.3 | 0.77 |
|  | C4 | 2.26E+02 | 6.06E+02 | 0.37 | 0.71 |
|  | C5 | -6.19E+01 | 1.69E+02 | -0.37 | 0.72 |
|  | C6 | 3.67E+01 | 1.70E+02 | 0.22 | 0.83 |
|  | C7 | -1.32E+02 | 2.25E+02 | -0.58 | 0.56 |
|  | C8 | -1.10E+02 | 1.66E+02 | -0.66 | 0.51 |
|  | C9 | -2.51E+01 | 2.01E+01 | -1.25 | 0.22 |
|  | C10 | 1.47E+01 | 1.34E+01 | 1.09 | 0.28 |
|  |  |  |  |  |  |
| NWR_length vs non-wordrepetition PRS | Intercept | 3.61E+01 | 7.54E+01 | 0.48 | 0.64 |
|  | Non-word repetition PRS | 1.10E+06 | 1.68E+06 | 0.65 | 0.52 |
|  | Age | 5.72E-02 | 1.10E+00 | 0.05 | 0.96 |
|  | Sex | -2.80E+00 | 2.60E+00 | -1.08 | 0.29 |
|  | Monogenic | -9.45E-01 | 2.81E+00 | -0.34 | 0.74 |
|  | C1 | 7.46E+03 | 6.28E+03 | 1.19 | 0.24 |
|  | C2 | 2.52E+03 | 1.74E+03 | 1.45 | 0.15 |
|  | C3 | -5.53E+02 | 6.12E+02 | -0.9 | 0.37 |
|  | C4 | 7.20E+02 | 1.03E+03 | 0.7 | 0.49 |
|  | C5 | -1.05E+02 | 2.88E+02 | -0.36 | 0.72 |
|  | C6 | -1.48E+02 | 2.90E+02 | -0.51 | 0.61 |
|  | C7 | -4.31E+02 | 3.85E+02 | -1.12 | 0.27 |
|  | C8 | -2.02E+02 | 2.83E+02 | -0.71 | 0.48 |
|  | C9 | -1.69E+01 | 3.43E+01 | -0.49 | 0.63 |
|  | C10 | -3.57E+00 | 2.29E+01 | -0.16 | 0.88 |
|  |  |  |  |  |  |
| NWR_PCC vs non-wordrepetition PRS | Intercept | 1.76E+01 | 2.78E+01 | 0.63 | 0.53 |
|  | Non-word repetition PRS | 2.35E+05 | 6.21E+05 | 0.38 | 0.71 |
|  | Age | -4.32E-01 | 4.06E-01 | -1.06 | 0.29 |
|  | Sex | 5.76E-01 | 9.57E-01 | 0.6 | 0.55 |
|  | Monogenic | -1.05E+00 | 1.04E+00 | -1.01 | 0.32 |
|  | C1 | 2.37E+03 | 2.31E+03 | 1.02 | 0.31 |
|  | C2 | 4.73E+02 | 6.42E+02 | 0.74 | 0.47 |
|  | C3 | -1.35E+02 | 2.25E+02 | -0.6 | 0.55 |
|  | C4 | 1.38E+02 | 3.81E+02 | 0.36 | 0.72 |
|  | C5 | -8.99E+01 | 1.06E+02 | -0.85 | 0.4 |
|  | C6 | 1.43E+01 | 1.07E+02 | 0.13 | 0.89 |
|  | C7 | -1.21E+02 | 1.42E+02 | -0.85 | 0.4 |
|  | C8 | -1.02E+02 | 1.04E+02 | -0.98 | 0.34 |
|  | C9 | -1.31E+01 | 1.26E+01 | -1.03 | 0.31 |
|  | C10 | 8.14E+00 | 8.44E+00 | 0.96 | 0.34 |
|  |  |  |  |  |  |
| TOWRE_words vs wordreading PRS | (Intercept) | -4.07E+00 | 1.24E+01 | -0.33 | 0.7435 |
|  | PRSwordreading | -2.15E+05 | 3.25E+05 | -0.66 | 0.5126 |
|  | age | 3.38E-01 | 1.87E-01 | 1.81 | 0.0775 |
|  | gender | 1.34E-01 | 4.29E-01 | 0.31 | 0.7565 |
|  | monogenic | -1.29E-01 | 4.61E-01 | -0.28 | 0.7812 |
|  | C1 | 7.83E+02 | 1.02E+03 | 0.77 | 0.4485 |
|  | C2 | 4.29E+02 | 2.86E+02 | 1.5 | 0.1411 |
|  | C3 | -7.95E+01 | 1.00E+02 | -0.79 | 0.4318 |
|  | C4 | 1.71E+02 | 1.70E+02 | 1.01 | 0.318 |
|  | C5 | -1.43E+02 | 4.72E+01 | -3.03 | 0.0041 |
|  | C6 | 1.04E+02 | 4.74E+01 | 2.19 | 0.0342 |
|  | C7 | -1.37E+01 | 6.37E+01 | -0.21 | 0.8314 |
|  | C8 | -6.40E+01 | 4.65E+01 | -1.38 | 0.1761 |
|  | C9 | -1.22E+01 | 5.70E+00 | -2.14 | 0.0381 |
|  | C10 | -2.78E+00 | 3.71E+00 | -0.75 | 0.458 |

**Supplementary references**

Abou Tayoun, A. N., Pesaran, T., DiStefano, M. T., Oza, A., Rehm, H. L., Biesecker, L. G., & Harrison, S. M. (2018). Recommendations for interpreting the loss of function PVS1 ACMG/AMP variant criterion. Human Mutation, 39(11). <https://doi.org/10.1002/humu.23626>

Demontis, D., Walters, G. B., Athanasiadis, G., Walters, R., Therrien, K., Nielsen, T. T., Farajzadeh, L., Voloudakis, G., Bendl, J., Zeng, B., Zhang, W., Grove, J., Als, T. D., Duan, J., Satterstrom, F. K., Bybjerg-Grauholm, J., Bækved-Hansen, M., Gudmundsson, O. O., Magnusson, S. H., … Børglum, A. D. (2023). Genome-wide analyses of ADHD identify 27 risk loci, refine the genetic architecture and implicate several cognitive domains. Nature Genetics 2023 55:2, 55(2), 198–208. <https://doi.org/10.1038/s41588-022-01285-8>

Drivas, T. G., Li, D., Nair, D., Alaimo, J. T., Alders, M., Altmüller, J., Barakat, T. S., Bebin, E. M., Bertsch, N. L., Blackburn, P. R., Blesson, A., Bouman, A. M., Brockmann, K., Brunelle, P., Burmeister, M., Cooper, G. M., Denecke, J., Dieux-Coëslier, A., Dubbs, H., … Bhoj, E. (2020). A second cohort of CHD3 patients expands the molecular mechanisms known to cause Snijders Blok-Campeau syndrome. European Journal of Human Genetics, 28(10), 1422–1431. <https://doi.org/10.1038/S41431-020-0654-4>

Eising, E., Carrion-Castillo, A., Vino, A., Strand, E. A., Jakielski, K. J., Scerri, T. S., Hildebrand, M. S., Webster, R., Ma, A., Mazoyer, B., Francks, C., Bahlo, M., Scheffer, I. E., Morgan, A. T., Shriberg, L. D., & Fisher, S. E. (2019). A set of regulatory genes co-expressed in embryonic human brain is implicated in disrupted speech development. Molecular Psychiatry, 24(7), 1065–1078. <https://doi.org/10.1038/S41380-018-0020-X>

Eising, E., Mirza-Schreiber, N., de Zeeuw, E. L., Wang, C. A., Truong, D. T., Allegrini, A. G., Shapland, C. Y., Zhu, G., Wigg, K. G., Gerritse, M. L., Molz, B., Alagoz, G., Gialluisi, A., Abbondanza, F., Rimfeld, K., van Donkelaar, M., Liao, Z., Jansen, P. R., Andlauer, T. F. M., … Fisher, S. E. (2022). Genome-wide analyses of individual differences in quantitatively assessed reading- and language-related skills in up to 34,000 people. Proceedings of the National Academy of Sciences of the United States of America, 119(35), e2202764119. <https://doi.org/10.1073/PNAS.2202764119/SUPPL_FILE/PNAS.2202764119.SD15.XLSX>

Grove, J., Ripke, S., Als, T. D., Mattheisen, M., Walters, R. K., Won, H., Pallesen, J., Agerbo, E., Andreassen, O. A., Anney, R., Awashti, S., Belliveau, R., Bettella, F., Buxbaum, J. D., Bybjerg-Grauholm, J., Bækvad-Hansen, M., Cerrato, F., Chambert, K., Christensen, J. H., … Børglum, A. D. (2019). Identification of common genetic risk variants for autism spectrum disorder. Nature Genetics, 51(3), 431. <https://doi.org/10.1038/S41588-019-0344-8>

Gelfman, S., Wang, Q., McSweeney, K. M., Ren, Z., la Carpia, F., Halvorsen, M., Schoch, K., Ratzon, F., Heinzen, E. L., Boland, M. J., Petrovski, S., & Goldstein, D. B. (2017). Annotating pathogenic non-coding variants in genic regions. Nature Communications, 8(1), 1–11. <https://doi.org/10.1038/s41467-017-00141-2>

Jaganathan, K., Kyriazopoulou Panagiotopoulou, S., McRae, J. F., Darbandi, S. F., Knowles, D., Li, Y. I., Kosmicki, J. A., Arbelaez, J., Cui, W., Schwartz, G. B., Chow, E. D., Kanterakis, E., Gao, H., Kia, A., Batzoglou, S., Sanders, S. J., & Farh, K. K. H. (2019). Predicting Splicing from Primary Sequence with Deep Learning. Cell, 176(3), 535-548.e24. <https://doi.org/10.1016/j.cell.2018.12.015>

Lee, J. J., Wedow, R., Okbay, A., Kong, E., Maghzian, O., Zacher, M., Nguyen-Viet, T. A., Bowers, P., Sidorenko, J., Karlsson Linnér, R., Fontana, M. A., Kundu, T., Lee, C., Li, H., Li, R., Royer, R., Timshel, P. N., Walters, R. K., Willoughby, E. A., … Turley, P. (2018). Gene discovery and polygenic prediction from a genome-wide association study of educational attainment in 1.1 million individuals. Nature Genetics, 50(8), 1112–1121. <https://doi.org/10.1038/S41588-018-0147-3>

Okbay, A., Wu, Y., Wang, N., Jayashankar, H., Bennett, M., Nehzati, S. M., Sidorenko, J., Kweon, H., Goldman, G., Gjorgjieva, T., Jiang, Y., Hicks, B., Tian, C., Hinds, D. A., Ahlskog, R., Magnusson, P. K. E., Oskarsson, S., Hayward, C., Campbell, A., … Young, A. I. (2022). Polygenic prediction of educational attainment within and between families from genome-wide association analyses in 3 million individuals. Nature Genetics 2022 54:4, 54(4), 437–449. <https://doi.org/10.1038/s41588-022-01016-z>

Pejaver, V., Byrne, A. B., Feng, B. J., Pagel, K. A., Mooney, S. D., Karchin, R., O’Donnell-Luria, A., Harrison, S. M., Tavtigian, S. V., Greenblatt, M. S., Biesecker, L. G., Radivojac, P., Brenner, S. E., Tayoun, A. A., Berg, J. S., Cutting, G. R., Ellard, S., Kang, P., Karbassi, I., … Topper, S. (2022). Calibration of computational tools for missense variant pathogenicity classification and ClinGen recommendations for PP3/BP4 criteria. American Journal of Human Genetics, 109(12). <https://doi.org/10.1016/j.ajhg.2022.10.013>

van der Spek, J., den Hoed, J., Snijders Blok, L., Dingemans, A. J. M., Schijven, D., Nellaker, C., Venselaar, H., Astuti, G. D. N., Barakat, T. S., Bebin, E. M., Beck-Wödl, S., Beunders, G., Brown, N. J., Brunet, T., Brunner, H. G., Campeau, P. M., Čuturilo, G., Gilissen, C., Haack, T. B., … Kleefstra, T. (2022). Inherited variants in CHD3 show variable expressivity in Snijders Blok-Campeau syndrome. Genetics in Medicine, 24(6), 1283–1296. <https://doi.org/10.1016/J.GIM.2022.02.014>

Weiner, D. J., Wigdor, E. M., Ripke, S., Walters, R. K., Kosmicki, J. A., Grove, J., Samocha, K. E., Goldstein, J. I., Okbay, A., Bybjerg-Grauholm, J., Werge, T., Hougaard, D. M., Taylor, J., Skuse, D., Devlin, B., Anney, R., Sanders, S. J., Bishop, S., Mortensen, P. B., … Arking, D. E. (2017). Polygenic transmission disequilibrium confirms that common and rare variation act additively to create risk for autism spectrum disorders. Nature Genetics, 49(7), 978. <https://doi.org/10.1038/NG.3863>
